# Predicted antiviral drugs Darunavir, Amprenavir, Rimantadine and Saquinavir can potentially bind to neutralize SARS-CoV-2 conserved proteins

**DOI:** 10.1186/s40709-021-00149-2

**Published:** 2021-08-04

**Authors:** Umesh C. Halder

**Affiliations:** Department of Zoology, Raniganj Girls’ College, Searsole -Rajbari, Paschim Bardhaman, Raniganj, 713358 West Bengal India

**Keywords:** SARS-CoV-2, COVID-19, Antiviral drugs, Darunavir, Amprenavir, Rimantadine, Saquinavir, Non-structural proteins, Enzymes

## Abstract

**Background:**

Novel Coronavirus disease 2019 or COVID-19 has become a threat to human society due to fast spreading and increasing mortality. It uses vertebrate hosts and presently deploys humans. Life cycle and pathogenicity of SARS-CoV-2 have already been deciphered and possible drug target trials are on the way.

**Results:**

The present study was aimed to analyze Non-Structural Proteins that include conserved enzymes of SARS-CoV-2 like papain-like protease, main protease, Replicase, RNA-dependent RNA polymerase, methyltransferase, helicase, exoribonuclease and endoribonucleaseas targets to all known drugs. A bioinformatic based web server Drug ReposeER predicted several drug binding motifs in these analyzed proteins. Results revealed that anti-viral drugs Darunavir,Amprenavir, Rimantadine and Saquinavir were the most potent to have 3D-drug binding motifs that were closely associated with the active sites of the SARS-CoV-2 enzymes .

**Conclusions:**

Repurposing of the antiviral drugs Darunavir, Amprenavir, Rimantadine and Saquinavir to treat COVID-19 patients could be useful that can potentially prevent human mortality.

**Graphic abstract:**

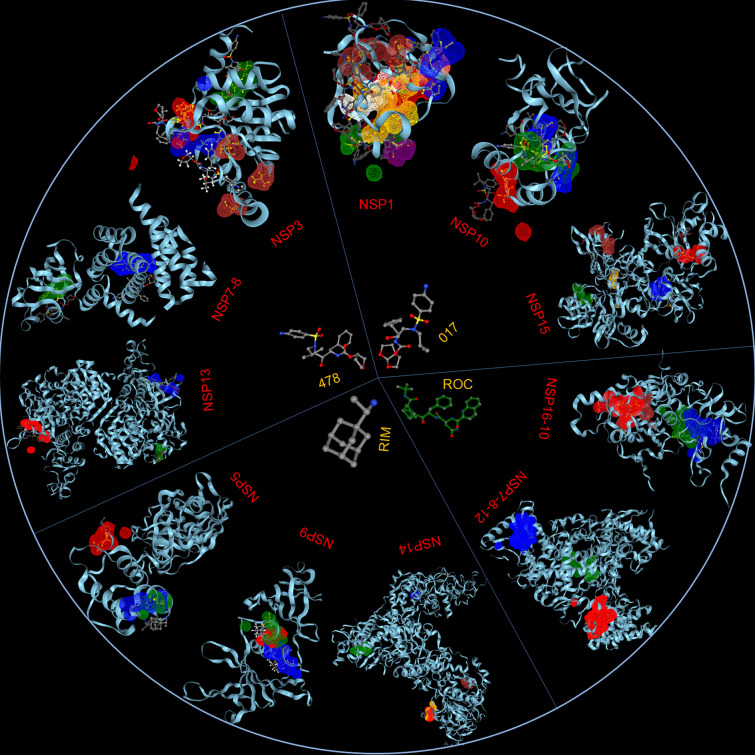

**Supplementary Information:**

The online version contains supplementary material available at 10.1186/s40709-021-00149-2.

## Background

SARS-CoV-2 has become a menace to the humanity and it imposed unprecedented epidemic condition. Great efforts were carried out by the scientists to develop potent vaccines like Astrazeneca/Oxford [1], Johnson & Johnson [2], Moderna [3], Pfizer/BionTech [4], Sinopharm, Sinovac [5], and COVISHIELD [6], having the potential to curb human mortality. The virus (a positive sense RNA virus with a genome of ~ 30 kb) has several types of vertebrate hosts including humans and transmission occurs through direct contact or aerosols [7, 8]. Like all animal viruses, their proteins hijack the cellular machineries to complete life cycle. These proteins are of great interest to the scientists to develop specific drug(s) or vaccine schemes against them. Search and trial of potential inhibitory drugs such as Remdesivir, Lopinavir-Ritonaviris were on the way but they were proven ineffective to prevent patient death [9–11]. The present work is based on the fact that most of the viral non-structural proteins (NSPs) which include enzymes remain structurally and chemically conserved as they have to interact with human proteins to carry out same biochemical processes within cell. SARS-CoV-2 genome encodes 16 non-structural proteins (NSPs), involved in genome replication and transcription [12, 13]. Nsp1 is a transcriptional, translational inhibitor and evades host immune system [14–16]. Nsp2 is involved in viral replication, disrupts host cell environment and, along with Nsp3, form proteases [12, 13]. Nsp4 interacts with Nsp3 to mediate viral replication [12, 13]. Main protease(M^pro^) or NSP5 is essential for viral replication [7, 8, 12, 13]. Nsp6 generate autophagosomes that assemble replicase proteins [12, 13]. Nsp7, Nsp8 and Nsp12 form RNA polymerase complex [17, 18]. NSP9 replicase is dimeric and involved in viral RNA synthesis [7, 8, 12, 13, 19]. Nsp10 stimulate Nsp14 and Nsp16 which are methyl transferases [14, 20]. The function of Nsp11 is yet to be deciphered [12, 13]. Nsp13 together with Nsp12 exert helicase activity and is involved in capping of viral RNA [21]. Nsp14 has exoribonuclease and N7-methyltransferase activity [22]. Coronavirus endoribonuclease (NSP15/EndoU) is a hexameric protein that preferentially recognizes and cleaves RNA [7, 8, 12, 13, 23] and EndoU also evades host mediated viral double-stranded RNA recognition. Nsp16 has methyltransferase activity and complexes with Nsp10 [7, 8, 12, 13, 24].

In the present study, 11 PDB entries (7K3N, 6WEY, 6M03, 7JLT, 6W4B, 6ZCT, 6M71, 7NIO, 5C8S, 6VWW and 7BQ7) [25–35] representing twelve non-structural proteins and their complexes of SARS-CoV-2, i.e., NSP1, NSP3,NSP5, NSP7-8 complex, NSP9, NSP10, NSP7-8–12 complex, NSP13, NSP14, NSP15 and NSP16-10 complex respectively have been analyzed using Drug ReposeER web server program (http://27.126.156.175/drreposed/) [36] for their possible binding sites [37] to all drugs available in drug bank. Only the NSPs having 3D structures available in PDB, have been considered in the study as tertiary structures have utmost requirement to find 3D drug binding interfaces. The drug binding interfaces showed congruence with the known drug binding motifs (Additional file 1: S1, Additional file 2: S2, Additional file 3: S4, Additional file 4: S4, Additional file 5: S5, Additional file 6: S6, Additional file 7: S7, Additional file 8: S8, Additional file 9: S9, Additional file 10: S10 and Additional file 11: S11) .

## Results and discussion

DrReposER predicted numerous potential 3D-drug binding motifs of both left (L) and right (R) superpositions for 7K3N, 6WEY, 6M03, 7JLT, 6W4B, 6ZCT, 6M71, 7NIO, 5C8S, 6VWW and 7BQ7 (Additional file 1: S1, Additional file 2: S2, Additional file 3: S4, Additional file 4: S4, Additional file 5: S5, Additional file 6: S6, Additional file 7: S7, Additional file 8: S8, Additional file 9: S9, Additional file 10: S10 and Additional file 11: S11). Known drugs that bind these motifs bind either human, bacterial or viral proteins. Results after analyzing the 3D structures of the target molecules and complexes were further filtered for anti-viral drugs. From the hit results, 14 anti-viral drugs i.e., Amphetamine (Drug bank ID-DB00182), Amprenavir (Drug bank ID-DB00701), Atazanavir (Drug bank ID-DB01072), Darunavir (Drug bank ID-DB01264), Grazoprevir (Drug bank ID-DB11575), Indinavir (Drug bank ID-DB00224), Lopinavir (Drug bank ID-DB01601), Nelfinavir (Drug bank ID-DB00220), Nevirapine (Drug bank ID-DB00238), Ribavirin (Drug bank ID-DB00811), Rimantadine (Drug bank ID-DB00478), Ritonavir (Drug bank ID-DB00503), Saquinavir (Drug bank ID-DB01232), and Tipranavir (Drug bank ID-DB00932) were selected for having unique 3D-drug binding motifs (Tables 1, 2, 3, 4, 5, 6, 7, 8, 9, 10 and 11). The findings showed that several anti-viral drugs had binding interfaces on a single protein or protein complexes and moreover, each anti-viral drug had one to several binding motifs (Tables 12 and 13).

**Table 1:** Possible binding sites of NSP1 against known anti-viral drugs

Drugs		Total binding sites				(7K3N) NSP1 of COVID-19
Amprenavir	Known similar target molecule					Protease, HIV-1
	Binding properties	3		1	Superposition type	R
					RMSD	0.91 Å
					Amino acid targets of drug	85GLY 86 ILE 58 PRO
					No. of residues in known binding	24
					Human similar targets	4
				2	Superposition type	L
					RMSD	0.89 Å
					Amino acid targets of drug	105 ILE 103 GLY 102 VAL
					No. of residues in known binding	25
					Human similar targets	4
				3	Superposition type	L
					RMSD	0.94 Å
					Amino acid targets of drug	24 ASP 83 LEU 97 VAL
					No. of residues in known binding	28
					Human similar targets	13
Atazanavir	Known similar target molecule					Protease, HIV-1
	Binding properties	1		1	Superposition type	R
					RMSD	0.98 Å
					Amino acid targets of drug	105 ILE 103 GLY 102 VAL
					No. of residues in known binding	24
					Human similar targets	5
Darunavir	Known similar target molecule					Pol polyprotein, HIV-2
	Binding properties	10	1		Superposition type	L
					RMSD	0.91 Å
					Amino acid targets of drug	105 ILE 103 GLY 102 VAL
					No. of residues in known binding	27
					Human similar targets	6
			2		Superposition type	L
					RMSD	0.89 Å
					Amino acid targets of drug	85 GLY 86 ILE 58 PRO
					No. of residues in known binding	26
					Human similar targets	0
			3		Superposition type	R
					RMSD	1.47 Å
					Amino acid targets of drug	98 LEU 29 VAL 99 VAL
					No. of residues in known binding	20
					Human similar targets	6
			4		Superposition type	L
					RMSD	1.19 Å
					Amino acid targets of drug	95 LEU 80 VAL 77 VAL
					No. of residues in known binding	20
					Human similar targets	6
			5		Superposition type	L
					RMSD	1.40 Å
					Amino acid targets of drug	79 LEU 26 VAL 60 VAL
					No. of residues in known binding	20
					Human similar targets	6
			6		Superposition type	L
					RMSD	1.32 Å
					Amino acid targets of drug	44 LEU 14 VAL 97 VAL
					No. of residues in known binding	20
					Human similar targets	6
			7		Superposition type	L
					RMSD	1.16 Å
					Amino acid targets of drug	83 LEU 60 VAL 26 VAL
					No. of residues in known binding	20
					Human similar targets	6
			8		Superposition type	L
					RMSD	1.47 Å
					Amino acid targets of drug	98 LEU 11 VAL 97 VAL
					No. of residues in known binding	20
					Human similar targets	6
			9		Superposition type	L
					RMSD	1.11 Å
					Amino acid targets of drug	55 LEU 60 VAL 99 VAL
					No. of residues in known binding	20
					Human similar targets	6
			10		Superposition type	L
					RMSD	1.36 Å
					Amino acid targets of drug	18 LEU 99 VAL 102 VAL
					No. of residues in known binding	20
					Human similar targets	6
Indinavir	Known similar target molecule					Protease retropepsin, HIV-1
	Binding properties	1		1	Superposition type	R
					RMSD	0.86 Å
					Amino acid targets of drug	47 VAL 96 GLY 62 ILE
					No. of residues in known binding	21
					Human similar targets	3
Nelfinavir	Known similar target molecule					Protease, HIV-1
	Binding properties	2		1	Superposition type	L
					RMSD	1.16 Å
					Amino acid targets of drug	110 ARG 95 LEU 75 VAL
					No. of residues in known binding	30
					Human similar targets	10
				2	Superposition type	L
					RMSD	1.49 Å
					Amino acid targets of drug	20 ARG 55 LEU 14 VAL
					No. of residues in known binding	30
					Human similar targets	10
Rimantadine	Known similar target molecule					M2 protein, Influenza A/B
	Binding properties	1		1	Superposition type	R
					RMSD	1.10 Å
					Amino acid targets of drug	29 VAL 33 ALA 31 SER
					No. of residues in known binding	10
					Human similar targets	0
Saquinavir	Known similar target molecule					Protease, HIV-1
	Binding properties	2		1	Superposition type	R
					RMSD	1.31 Å
					Amino acid targets of drug	60 VAL 100 PRO 99 VAL 97 VAL
					No. of residues in known binding	22
					Human similar targets	6
				2	Superposition type	L
					RMSD	0.92 Å
					Amino acid targets of drug	105 ILE 103 GLY 102 VAL
					No. of residues in known binding	31
					Human similar targets	11
Tipranavir	Known similar target molecule					Protease, HIV-1
	Binding properties	1		1	Superposition type	L
					RMSD	0.87 Å
					Amino acid targets of drug	105 ILE 103 GLY 102 VAL
					No. of residues in known binding	27
					Human similar targets	3

Features of different drug binding motifs.* HIV-1* Human Immunodeficiency virus 1, *RMSD* Root Mean Square Deviation, *Å* angstrom, *ILE* isoleucine, *GLY* glycine, *VAL* valine, *LEU* leucine, *PRO* proline, *ASP* aspartic acid, *SER* serine

**Table 2:** Possible binding sites of NSP3 against known anti-viral drugs

Drugs		Total binding sites			(6WEY) NSP3 OF COVID-19
Amprenavir	Known similar target molecule				Protease, HIV-1
	Binding properties	4	1	Superposition type	R
				RMSD	1.13 Å
				Amino acid targets of drug	335 ILE 252 GLY 253 VAL
				No. of residues in known binding	25
				Human similar targets	2
			2	Superposition type	R
				RMSD	1.21 Å
				Amino acid targets of drug	335 ILE 337 GLY 304 VAL
				No. of residues in known binding	25
				Human similar targets	2
			3	Superposition type	R
				RMSD	1.01 Å
				Amino acid targets of drug	270 ASP 287 LEU 300 VAL
				No. of residues in known binding	28
				Human similar targets	11
			4	Superposition type	R
				RMSD	0.88 Å
				Amino acid targets of drug	214 LEU 359 VAL 222 ILE
				No. of residues in known binding	18
				Human similar targets	5
Darunavir	Known similar target molecule				Protease, HIV-1
	Binding properties	6	1	Superposition type	R
				RMSD	1.03 Å
				Amino acid targets of drug	335 ILE 252 GLY 253 VAL
				No. of residues in known binding	27
				Human similar targets	6
			2	Superposition type	L
				RMSD	0.97 Å
				Amino acid targets of drug	216 LEU 355 VAL 348 VAL
				No. of residues in known binding	20
				Human similar targets	5
			3	Superposition type	L
				RMSD	1.18 Å
				Amino acid targets of drug	297 LEU 355 VAL 240 VAL
				No. of residues in known binding	20
				Human similar targets	6
			4	Superposition type	R
				RMSD	0.93 Å
				Amino acid targets of drug	231 ALA 227 ILE 239 VAL
				No. of residues in known binding	19
				Human similar targets	13
			5	Superposition type	R
				RMSD	0.86 Å
				Amino acid targets of drug	292 LEU 234 VAL 239 VAL
				No. of residues in known binding	20
				Human similar targets	7
			6	Superposition type	R
				RMSD	1.28 Å
				Amino acid targets of drug	287 LEU 240 VAL 286 VAL
				No. of residues in known binding	20
				Human similar targets	7
Rimantadine	Known similar target molecule				M2 protein, Influeza A
	Binding properties	2	1	Superposition type	L
				RMSD	0.94 Å
				Amino acid targets of drug	333 ALA 332 SER 337 GLY
				No. of residues in known binding	9
				Human similar targets	0
			2	Superposition type	R
				RMSD	1.08 Å
				Amino acid targets of drug	281 VAL 316 ALA 315 SER
				No. of residues in known binding	9
				Human similar targets	0
Saquinavir	Known similar target molecule				Protease, HIV-1
	Binding properties	1	1	Superposition type	R
				RMSD	1.25 Å
				Amino acid targets of drug	335 ILE 252 GLY 253 VAL
				No. of residues in known binding	31
				Human similar targets	12
Tipranavir	Known similar target molecule				Protease, HIV-1
	Binding properties	2	1	Superposition type	R
				RMSD	1.14 Å
				Amino acid targets of drug	335 ILE 337 GLY 304 VAL
				No. of residues in known binding	27
				Human similar targets	3
			2	Superposition type	R
				RMSD	1.10 Å
				Amino acid targets of drug	335 ILE 252 GLY 253 VAL
				No. of residues in known binding	27
				Human similar targets	3

Features of different drug binding motifs. *HIV-1* Human Immunodeficiency virus 1, *RMSD* Root Mean Square Deviation, *Å* angstrom, *ILE* isoleucine, *GLY* glycine, *VAL* valine, *LEU* leucine, *PRO* proline, *ASP* aspartic acid, *SER* serine

**Table 3:** Possible binding sites of NSP5 against known anti-viral drugs.

Drugs		Total binding sites			(6M03) NSP5 of COVID-19
Amphetamine	Known similar target molecule				Polymerase polyprotein, HIV-1
	Binding properties	1	1	Superposition type	L
				RMSD	0.93 Å
				Amino acid targets of drug	122 PRO 120 GLY 28 ASN
				No. of residues in known binding	16
				Human similar targets	0
Darunavir	Known similar target molecule				HIV-1 protease
	Binding properties	2	1	Superposition type	L
				RMSD	1.06 Å
				Amino acid targets of drug	109 GLY 200 ILE 293 PRO
				No. of residues in known binding	26
				Human similar targets	0
			2	Superposition type	R
				RMSD	0.76 Å
				Amino acid targets of drug	133 ASN 195 GLY 194 ALA
				No. of residues in known binding	26
				Human similar targets	2
Indinavir	Known similar target molecule				Protease retropepsin, HIV-1
	Binding properties	1	1	Superposition type	L
				RMSD	0.81 Å
				Amino acid targets of drug	106 ILE 109 GLY 200 ILE
				No. of residues in known binding	22
				Human similar targets	4
Nelfinavir	Known similar target molecule				Protease retropepsin, HIV-1
	Binding properties	1	1	Superposition type	L
				RMSD	1.05 Å
				Amino acid targets of drug	153 ASP 292 THR 293 PRO
				No. of residues in known binding	30
				Human similar targets	13
Nevirapine	Known similar target molecule				Reverse transcriptase, HIV-1
	Binding properties	1	1	Superposition type	R
				RMSD	1.10 Å
				Amino acid targets of drug	88 LYS 86 VAL 30 LEU
				No. of residues in known binding	7
				Human similar targets	13
Ribavirin	Known similar target molecule				RNA polymerase, Norwalk virus
	Binding properties	1	1	Superposition type	L
				RMSD	1.06 Å
				Amino acid targets of drug	198 THR 199 THR 238 ASN
				No. of residues in known binding	9
				Human similar targets	2
Rimantadine	Known similar target molecule				M2 protein, Influenza A
	Binding properties	3	1	Superposition type	R
				RMSD	0.95 Å
				Amino acid targets of drug	255 ALA 254 SER 251 GLY
				No. of residues in known binding	9
				Human similar targets	0
			2	Superposition type	L
				RMSD	0.88 Å
				Amino acid targets of drug	255 ALA 254 SER 258 GLY
				No. of residues in known binding	9
				Human similar targets	0
			3	Superposition type	L
				RMSD	1.00 Å
				Amino acid targets of drug	285 ALA 284 SER 283 GLY
				No. of residues in known binding	9
				Human similar targets	0
Ritonavir	Known similar target molecule				Polymerase polyprotein, HIV-1
	Binding properties	1	1	Superposition type	L
				RMSD	0.82 Å
				Amino acid targets of drug	106 ILE 109 GLY 200 ILE
				No. of residues in known binding	18
				Human similar targets	4
Tipranavir	Known similar target molecule				Protease, HIV-1
	Binding properties	1	1	Superposition type	L
				RMSD	1.17 Å
				Amino acid targets of drug	94 ALA 34 ASP 33 ASP
				No. of residues in known binding	27
				Human similar targets	3

Features of different drug binding motifs. *HIV-1* Human Immunodeficiency virus 1, *RMSD* Root Mean Square Deviation, *Å* angstrom, *ILE* isoleucine, *GLY* glycine, *VAL* valine, *LEU* leucine, *PRO* proline, *ASP* aspartic acid, *ASN* asparagine, *ALA* alanine, *THR* threonine, *LYS* lysine, *SER* serine

**Table 4:** Possible binding sites of NSP7-NSP8 against known anti-viral drugs

Drugs		Total binding sites			(7JLT) NSP7-NSP8 of COVID-19
Amprenavir	Known similar target molecule				Protease, HIV-1
	Binding properties	2	1	Superposition type	L
				RMSD	1.09 Å
				Amino acid targets of drug	184 LEU 130 VAL 132 ILE
				No. of residues in known binding	18
				Human similar targets	5
			2	Superposition type	R
				RMSD	1.07 Å
				Amino acid targets of drug	13 LEU 11 VAL 16 VAL 12 VAL
				No. of residues in known binding	18
				Human similar targets	5
Darunavir	Known similar target molecule				Protease, HIV-1
	Binding properties	1	1	Superposition type	R
				RMSD	0.99 Å
				Amino acid targets of drug	13 LEU 11 VAL 16 VAL
				No. of residues in known binding	20
				Human similar targets	6
Nelfinavir	Known similar target molecule				Protease, HIV-1
	Binding properties	1	1	Superposition type	R
				RMSD	0.92 Å
				Amino acid targets of drug	77 ASP 78 ASN 93 THR
				No. of residues in known binding	30
				Human similar targets	10
Rimantadine	Known similar target molecule				M2 protein, Influeza A
	Binding properties	1	1	Superposition type	L
				RMSD	0.96 Å
				Amino acid targets of drug	83 VAL 86 ALA 85 SER
				No. of residues in known binding	10
				Human similar targets	0
Saquinavir	Known similar target molecule				Protease, HIV-1
	Binding properties	1	1	Superposition type	R
				RMSD	0.97 Å
				Amino acid targets of drug	160 VAL 183 PRO 185 ILE
				No. of residues in known binding	31
				Human similar targets	5

Features of different drug binding motifs. *HIV-1* Human Immunodeficiency virus 1, *RMSD* Root Mean Square Deviation, *Å* angstrom, *ILE* isoleucine, *GLY* glycine, *VAL* valine, *LEU* leucine, *PRO* proline, *ASP* aspartic acid

**Table 5:** Possible binding sites of NSP9 against known anti-viral drugs

Drugs		Total binding sites			(6W4B) NSP9 Replicase of COVID-19
Grazoprevir	Known similar target molecule				NS3 protease, NS4a protein, Hepacivirus C
	Binding properties	1	1	Superposition type	L
				RMSD	0.94 Å
				Amino acid targets of drug	66 ILE 59 LYS 62 GLY
				No. of residues in known binding	16
				Human similar targets	8
Ribavirin	Known similar target molecule				RNA polymerase, Norwalk virus
	Binding properties	1	1	Superposition type	R
				RMSD	0.80 Å
				Amino acid targets of drug	36 THR 35 THR 34 ASN
				No. of residues in known binding	09
				Human similar targets	2
Rimantadine	Known similar target molecule				M2, BM2 protein, Influenza A,B
	Binding properties	3	1	Superposition type	L
				RMSD	0.90 Å
				Amino acid targets of drug	109 ALA 106 SER 105 GLY
				No. of residues in known binding	9
				Human similar targets	0
			2	Superposition type	R
				RMSD	1.10 Å
				Amino acid targets of drug	111 VAL 108 VAL 106 SER
				No. of residues in known binding	10
				Human similar targets	0
			3	Superposition type	R
				RMSD	1.26 Å
				Amino acid targets of drug	111 VAL 109 ALA 106 SER
				No. of residues in known binding	10
				Human similar targets	0
Tipranavir	Known similar target molecule				Protease, HIV-1
	Binding properties	1	1	Superposition type	L
				RMSD	1.21 Å
				Amino acid targets of drug	16 ALA 26 ASP 27 ASP
				No. of residues in known binding	27
				Human similar targets	3

Features of different drug binding motifs. *HIV-1* Human Immunodeficiency virus 1, *RMSD* Root Mean Square Deviation, *Å* angstrom, *ILE* isoleucine, *GLY* glycine, *VAL* valine, *ASP* aspartic acid, *ASN* asparagine, *ALA* alanine, *THR* threonine, *LYS* lysine, *SER* serine

**Table 6:** Possible binding sites of NSP10 against known anti-viral drugs

Drugs		Total binding sites			(6ZCT) NSP10 of COVID-19
Atazanavir	Known similar target molecule				Protease, HIV-1
	Binding properties	2	1	Superposition type	R
				RMSD	0.94 Å
				Amino acid targets of drug	107 PRO 108 VAL 38 ILE
				No. of residues in known binding	19
				Human similar targets	4
			2	Superposition type	L
				RMSD	1.01 Å
				Amino acid targets of drug	78 ARG 107 PRO 108 VAL
				No. of residues in known binding	23
				Human similar targets	3
Darunavir	Known similar target molecule				Protease, HIV-1
	Binding properties	3	1	Superposition type	R
				RMSD	0.99 Å
				Amino acid targets of drug	78 ARG 107 PRO 108 VAL
				No. of residues in known binding	26
				Human similar targets	7
			2	Superposition type	L
				RMSD	1.09 Å
				Amino acid targets of drug	78 ARG 37 PRO 38 ILE
				No. of residues in known binding	22
				Human similar targets	6
			3	Superposition type	L
				RMSD	0.85 Å
				Amino acid targets of drug	26 ALA 22 ASP 21 VAL
				No. of residues in known binding	27
				Human similar targets	8
Grazoprevir	Known similar target molecule				NS3, NS4 Protease, Hepacivirus C
	Binding properties	2	1	Superposition type	L
				RMSD	0.76 Å
				Amino acid targets of drug	65 GLN 52 GLY 127 GLY
				No. of residues in known binding	17
				Human similar targets	8
			2	Superposition type	L
				RMSD	0.88 Å
				Amino acid targets of drug	36 GLN 35 GLY 9 GLY
				No. of residues in known binding	17
				Human similar targets	8
Indinavir	Known similar target molecule				Polyprotein, HIV-1
	Binding properties	1	1	Superposition type	L
				RMSD	0.94 Å
				Amino acid targets of drug	78 ARG 107 PRO 108 VAL
				No. of residues in known binding	24
				Human similar targets	2
Lopinavir	Known similar target molecule				Protease, HIV-1
	Binding properties	1	1	Superposition type	L
				RMSD	0.84 Å
				Amino acid targets of drug	78 ARG 107 PRO 108 VAL
				No. of residues in known binding	23
				Human similar targets	6
Ritonavir	Known similar target molecule				Protease, HIV-1
	Binding properties	1	1	Superposition type	L
				RMSD	1.12 Å
				Amino acid targets of drug	78 ARG 107 PRO 108 VAL
				No. of residues in known binding	23
				Human similar targets	4
Saquinavir	Known similar target molecule				Protease, HIV-1
	Binding properties	1	1	Superposition type	L
				RMSD	0.91 Å
				Amino acid targets of drug	78 ARG 107 PRO 108 VAL
				No. of residues in known binding	31
				Human similar targets	8

Features of different drug binding motifs. *HIV-1* Human Immunodeficiency virus 1, *RMSD* Root Mean Square Deviation, *Å* angstrom, *ILE* isoleucine, *GLY* glycine, *VAL* valine, *LEU* leucine, *PRO* proline, *ASP* aspartic acid

**Table 7:** Possible binding sites of NSP7-NSP8-NSP12 complex against known anti-viral drugs

Drugs		Total binding sites			(6M71) NSP7-NSP8-NSP12 complex of COVID-19
Amprenavir	Known similar target molecule				Protease, HIV-1
	Binding properties	3	1	Superposition type	L
				RMSD	0.78 Å
				Amino acid targets of drug	223 ILE 203 GLY 204 VAL
				No. of residues in known binding	25
				Human similar targets	4
			2	Superposition type	R
				RMSD	0.66 Å
				Amino acid targets of drug	201 ILE 203 GLY 204 VAL
				No. of residues in known binding	25
				Human similar targets	5
			3	Superposition type	L
				RMSD	0.89 Å
				Amino acid targets of drug	760 ASP 786 LEU 166 VAL
				No. of residues in known binding	28
				Human similar targets	11
	Known similar target molecule				Protease, HIV-1
Atazanavir	Binding properties	1	1	Superposition type	R
				RMSD	0.69 Å
				Amino acid targets of drug	201 ILE 203 GLY 204 VAL
				No. of residues in known binding	24
				Human similar targets	4
Darunavir	Known similar target molecule				Protease, HIV-1
	Binding properties	6	1	Superposition type	L
				RMSD	0.64 Å
				Amino acid targets of drug	223 ILE 203 GLY 204 VAL
				No. of residues in known binding	27
				Human similar targets	6
			2	Superposition type	R
				RMSD	0.69 Å
				Amino acid targets of drug	201 ILE 203 GLY 204 VAL
				No. of residues in known binding	27
				Human similar targets	6
			3	Superposition type	R
				RMSD	0.90 Å
				Amino acid targets of drug	103 LEU 119 ILE 107 ILE
				No. of residues in known binding	22
				Human similar targets	12
			4	Superposition type	R
				RMSD	0.74 Å
				Amino acid targets of drug	102 ALA 106 ILE 53 VAL
				No. of residues in known binding	19
				Human similar targets	12
Indinavir	Known similar target molecule				Polyprotein, HIV-1
	Binding properties	1	1	Superposition type	L
				RMSD	0.87 Å
				Amino acid targets of drug	201 ILE 200 GLY 230 GLY
				No. of residues in known binding	22
				Human similar targets	7
Nelfinavir	Known similar target molecule				Protease, HIV-1
	Binding properties	2	1	Superposition type	L
				RMSD	0.84 Å
				Amino acid targets of drug	358 ASP 534 ASN 567 THR
				No. of residues in known binding	30
				Human similar targets	10
			2	Superposition type	R
				RMSD	0.63 Å
				Amino acid targets of drug	631 ARG 663 LEU 662 VAL
				No. of residues in known binding	30
				Human similar targets	10
Rimantadine	Known similar target molecule				M2 protein, Influeza A
	Binding properties	1	1	Superposition type	R
				RMSD	0.91 Å
				Amino acid targets of drug	771 ALA 772 SER 774 GLY
				No. of residues in known binding	9
				Human similar targets	0
Saquinavir	Known similar target molecule				Protease, HIV-1
	Binding properties	3	1	Superposition type	L
				RMSD	0.73 Å
				Amino acid targets of drug	820 VAL 830 PRO 817 THR
				No. of residues in known binding	21
				Human similar targets	6
			2	Superposition type	R
				RMSD	0.91 Å
				Amino acid targets of drug	623 ASP 678 GLY 462 THR
				No. of residues in known binding	27
				Human similar targets	0
			3	Superposition type	R
				RMSD	0.61 Å
				Amino acid targets of drug	201 ILE 203 GLY 204 VAL
				No. of residues in known binding	31
				Human similar targets	11
Tipranavir	Known similar target molecule				Protease, HIV-1
	Binding properties	2	1	Superposition type	L
				RMSD	0.82 Å
				Amino acid targets of drug	223 ILE 203 GLY 204 VAL
				No. of residues in known binding	27
				Human similar targets	3
			2	Superposition type	R
				RMSD	0.58 Å
				Amino acid targets of drug	201 ILE 203 GLY 204 VAL
				No. of residues in known binding	27
				Human similar targets	3

Features of different drug binding motifs. *HIV-1* Human Immunodeficiency virus 1, *RMSD* Root Mean Square Deviation, *Å* angstrom, *ILE* isoleucine, *GLY* glycine, *VAL* valine, *LEU* leucine, *PRO* proline, *ASP* aspartic acid

**Table 8:** Possible binding sites of NSP13 against known anti-viral drugs

Drugs		Total binding sites			(7NIO) NSP13 of COVID-19
Amprenavir	Known similar target molecule				Protease, HIV-1
	Binding properties	3	1	Superposition type	R
				RMSD	0.81 Å
				Amino acid targets of drug	258 ILE 294 GLY 293 ILE
				No. of residues in known binding	24
				Human similar targets	6
			2	Superposition type	L
				RMSD	0.92 Å
				Amino acid targets of drug	151 ILE 184 GLY 195 ILE
				No. of residues in known binding	24
				Human similar targets	6
			3	Superposition type	L
				RMSD	0.76 Å
				Amino acid targets of drug	226 VAL 184 GLY 195 ILE
				No. of residues in known binding	18
				Human similar targets	16
Atazanavir	Known similar target molecule				Protease, HIV-1
	Binding properties	1	1	Superposition type	L
				RMSD	0.84 Å
				Amino acid targets of drug	258 ILE 294 GLY 293 ILE
				No. of residues in known binding	21
				Human similar targets	3
Darunavir	Known similar target molecule				Protease, HIV-1
	Binding properties	2	1	Superposition type	L
				RMSD	0.76 Å
				Amino acid targets of drug	258 ILE 294 GLY 293 ILE
				No. of residues in known binding	21
				Human similar targets	6
			2	Superposition type	L
				RMSD	0.72 Å
				Amino acid targets of drug	226 VAL 184 GLY 195 ILE
				No. of residues in known binding	22
				Human similar targets	12
Indinavir	Known similar target molecule				Polyprotein, HIV-1
	Binding properties	3	1	Superposition type	L
				RMSD	0.72 Å
				Amino acid targets of drug	226 VAL 184 GLY 195 ILE
				No. of residues in known binding	21
				Human similar targets	3
			2	Superposition type	R
				RMSD	0.92 Å
				Amino acid targets of drug	399 ILE 400 GLY 282 GLY
				No. of residues in known binding	22
				Human similar targets	7
			3	Superposition type	L
				RMSD	0.84 Å
				Amino acid targets of drug	258 ILE 294 GLY 293 ILE
				No. of residues in known binding	21
				Human similar targets	6
Lopinavir	Known similar target molecule				Protease, HIV-1
	Binding properties	2	1	Superposition type	L
				RMSD	0.84 Å
				Amino acid targets of drug	258 ILE 294 GLY 293 ILE
				No. of residues in known binding	27
				Human similar targets	4
			2	Superposition type	L
				RMSD	0.79 Å
				Amino acid targets of drug	282 GLY 400 GLY 376 ILE
				No. of residues in known binding	27
				Human similar targets	6
Nelfinavir	Known similar target molecule				Protease, HIV-1
	Binding properties	1	1	Superposition type	L
				RMSD	0.82 Å
				Amino acid targets of drug	258 ILE 294 GLY 293 ILE
				No. of residues in known binding	30
				Human similar targets	9
Rimantadine	Known similar target molecule				M2 protein, Influeza A
	Binding properties	2	1	Superposition type	L
				RMSD	0.88 Å
				Amino acid targets of drug	01 ALA 13 SER 03 GLY
				No. of residues in known binding	9
				Human similar targets	0
			2	Superposition type	R
				RMSD	0.84 Å
				Amino acid targets of drug	522 ALA 523 SER 527 GLY
				No. of residues in known binding	9
				Human similar targets	0
Ritonavir	Known similar target molecule				Protease, HIV-1
	Binding properties	1	1	Superposition type	L
				RMSD	0.82 Å
				Amino acid targets of drug	258 ILE 294 GLY 293 ILE
				No. of residues in known binding	18
				Human similar targets	4
Saquinavir	Known similar target molecule				Protease, HIV-1
	Binding properties	1	1	Superposition type	R
				RMSD	0.73 Å
				Amino acid targets of drug	258 ILE 294 GLY 293 ILE
				No. of residues in known binding	27
				Human similar targets	3
Tipranavir	Known similar target molecule				Protease, HIV-1
	Binding properties	1	1	Superposition type	L
				RMSD	0.87 Å
				Amino acid targets of drug	258 ILE 294 GLY 293 ILE
				No. of residues in known binding	27
				Human similar targets	7

Features of different drug binding motifs. *HIV-1* Human Immunodeficiency virus 1, *RMSD* Root Mean Square Deviation, *Å* angstrom, *ILE* isoleucine, *GLY* glycine, *VAL* valine, *LEU* leucine, *PRO* proline, *ASP* aspartic acid

**Table 9:** Possible binding sites of NSP14 against known anti-viral drugs

Drugs		Total binding sites			(5C8S) NSP14 of COVID-19
Amprenavir	Known similar target molecule				Protease, HIV-1
	Binding properties	3	1	Superposition type	R
				RMSD	0.83 Å
				Amino acid targets of drug	88 GLY 87 ILE 412 PRO
				No. of residues in known binding	24
				Human similar targets	4
			2	Superposition type	L
				RMSD	0.72 Å
				Amino acid targets of drug	170 LEU 162 VAL 166 ILE
				No. of residues in known binding	18
				Human similar targets	4
			3	Superposition type	L
				RMSD	0.87 Å
				Amino acid targets of drug	31 ILE 17 GLY 14 ILE
				No. of residues in known binding	24
				Human similar targets	6
Atazanavir	Known similar target molecule				Protease, HIV-1
	Binding properties	1	1	Superposition type	L
				RMSD	0.66 Å
				Amino acid targets of drug	78 ARG 107 PRO 108 VAL
				No. of residues in known binding	23
				Human similar targets	3
Darunavir	Known similar target molecule				Protease, HIV-1
	Binding properties	7	1	Superposition type	L
				RMSD	0.79 Å
				Amino acid targets of drug	88 GLY 87 ILE 412 PRO
				No. of residues in known binding	26
				Human similar targets	0
			2	Superposition type	L
				RMSD	1.38 Å
				Amino acid targets of drug	170 LEU 162 VAL 167 VAL 166 ILE
				No. of residues in known binding	26
				Human similar targets	7
			3	Superposition type	R
				RMSD	0.55 Å
				Amino acid targets of drug	78 ARG 107 PRO 108 VAL
				No. of residues in known binding	21
				Human similar targets	6
			4	Superposition type	L
				RMSD	0.79 Å
				Amino acid targets of drug	26 ALA 22 ASP 21 VAL
				No. of residues in known binding	27
				Human similar targets	7
			5	Superposition type	L
				RMSD	0.83 Å
				Amino acid targets of drug	435 ALA 390 ASP 389 VAL
				No. of residues in known binding	27
				Human similar targets	7
			6	Superposition type	R
				RMSD	0.94 Å
				Amino acid targets of drug	152 LEU 120 VAL 118 VAL
				No. of residues in known binding	20
				Human similar targets	6
			7	Superposition type	R
				RMSD	0.87 Å
				Amino acid targets of drug	508 LEU 317 VAL 312 VAL
				No. of residues in known binding	20
				Human similar targets	6
Grazoprevir	Known similar target molecule				NS3, NS4 Protease, Hepacivirus C
	Binding properties	1	1	Superposition type	L
				RMSD	0.90 Å
				Amino acid targets of drug	65 GLN 52 GLY 127 GLY
				No. of residues in known binding	17
				Human similar targets	7
Indinavir	Known similar target molecule				Polyprotein, HIV-1
	Binding properties	1	1	Superposition type	L
				RMSD	0.82 Å
				Amino acid targets of drug	78 ARG 107 PRO 108 VAL
				No. of residues in known binding	24
				Human similar targets	1
Lopinavir	Known similar target molecule				Protease, HIV-1
	Binding properties	2	1	Superposition type	R
				RMSD	0.65 Å
				Amino acid targets of drug	78 ARG 107 PRO 108 VAL
				No. of residues in known binding	27
				Human similar targets	6
			2	Superposition type	R
				RMSD	0.94 Å
				Amino acid targets of drug	31 ILE 17 GLY 14 ILE
				No. of residues in known binding	27
				Human similar targets	4
Rimantadine	Known similar target molecule				M2 protein, Influeza A
	Binding properties	5	1	Superposition type	L
				RMSD	0.86 Å
				Amino acid targets of drug	317 VAL 320 ALA 319 SER
				No. of residues in known binding	10
				Human similar targets	0
			2	Superposition type	L
				RMSD	0.90 Å
				Amino acid targets of drug	32 ALA 33 SER 34 GLY
				No. of residues in known binding	9
				Human similar targets	0
			3	Superposition type	L
				RMSD	0.80 Å
				Amino acid targets of drug	32 ALA 33 SER 35 GLY
				No. of residues in known binding	9
				Human similar targets	0
			4	Superposition type	R
				RMSD	0.67 Å
				Amino acid targets of drug	01 ALA 0 SER -1 GLY
				No. of residues in known binding	9
				Human similar targets	0
			5	Superposition type	R
				RMSD	0.91 Å
				Amino acid targets of drug	01 ALA 0 SER 102 GLY
				No. of residues in known binding	9
				Human similar targets	0
Saquinavir	Known similar target molecule				Protease, HIV-1
	Binding properties	2	1	Superposition type	L
				RMSD	0.90 Å
				Amino acid targets of drug	31 ILE 17 GLY 14 ILE
				No. of residues in known binding	21
				Human similar targets	4
			2	Superposition type	R
				RMSD	0.90 Å
				Amino acid targets of drug	84 ARG 244 VAL 277 THR
				No. of residues in known binding	29
				Human similar targets	9
Tipranavir	Known similar target molecule				Protease, HIV-1
	Binding properties	2	1	Superposition type	L
				RMSD	0.92 Å
				Amino acid targets of drug	274 ALA 273 ASP 90 ASP
				No. of residues in known binding	27
				Human similar targets	3
			2	Superposition type	L
				RMSD	1.25 Å
				Amino acid targets of drug	116 ASN 270 ALA 273 ASP 90 ASP
				No. of residues in known binding	18
				Human similar targets	2

Features of different drug binding motifs. *HIV-1* Human Immunodeficiency virus 1, *RMSD* Root Mean Square Deviation, *Å* angstrom, *ILE* isoleucine, *GLY* glycine, *VAL* valine, *LEU* leucine, *PRO* proline, *ASP* aspartic acid

**Table 10:** Possible binding sites of NSP15 against known anti-viral drugs

Drugs		Total binding sites			(6VWW) NSP15 endoribonuclease of COVID-19
Amprenavir	Known similar target molecule				Protease, HIV-1
	Binding properties	2	1	Superposition type	L
				RMSD	0.90 Å
				Amino acid targets of drug	72 ILE 157 GLY 156 VAL
				No. of residues in known binding	25
				Human similar targets	4
			2	Superposition type	L
				RMSD	0.66 Å
				Amino acid targets of drug	251 LEU 276 VAL 296 ILE
				No. of residues in known binding	19
				Human similar targets	5
Atazanavir	Known similar target molecule				Protease, HIV-1
	Binding properties	1	1	Superposition type	L
				RMSD	0.83 Å
				Amino acid targets of drug	72 ILE 157 GLY 156 VAL
				No. of residues in known binding	24
				Human similar targets	4
Darunavir	Known similar target molecule				Protease, HIV-2
	Binding properties	5	1	Superposition type	L
				RMSD	1.00 Å
				Amino acid targets of drug	3 LEU 23 VAL 6 VAL
				No. of residues in known binding	20
				Human similar targets	6
			2	Superposition type	L
				RMSD	0.95 Å
				Amino acid targets of drug	72 ILE 157 GLY 156 VAL
				No. of residues in known binding	20
				Human similar targets	10
			3	Superposition type	R
				RMSD	0.77 Å
				Amino acid targets of drug	73 LEU 80 ILE 86 ILE
				No. of residues in known binding	22
				Human similar targets	12
			4	Superposition type	R
				RMSD	0.91 Å
				Amino acid targets of drug	300 LEU 212 ILE 253 ILE
				No. of residues in known binding	22
				Human similar targets	12
			5	Superposition type	L
				RMSD	0.77 Å
				Amino acid targets of drug	300 LEU 296 ILE 253 ILE
				No. of residues in known binding	22
				Human similar targets	12
Indinavir	Known similar target molecule				Protease retropepsin, HIV-1
	Binding properties	3	1	Superposition type	R
				RMSD	0.99 Å
				Amino acid targets of drug	122 VAL 119 PRO 80 ILE
				No. of residues in known binding	21
				Human similar targets	6
			2	Superposition type	R
				RMSD	0.87 Å
				Amino acid targets of drug	173 VAL 170 GLY 169 ILE
				No. of residues in known binding	21
				Human similar targets	3
			3	Superposition type	L
				RMSD	0.96 Å
				Amino acid targets of drug	321 VAL 344 PRO 323 ILE
				No. of residues in known binding	21
				Human similar targets	6
Lopinavir	Known similar target molecule				Protease, HIV-1
	Binding properties	3	1	Superposition type	R
				RMSD	0.90 Å
				Amino acid targets of drug	122 VAL 119 PRO 80 ILE
				No. of residues in known binding	23
				Human similar targets	6
			2	Superposition type	R
				RMSD	0.80 Å
				Amino acid targets of drug	247 GLY 248 GLY 236 ILE
				No. of residues in known binding	27
				Human similar targets	6
			3	Superposition type	L
				RMSD	0.89 Å
				Amino acid targets of drug	321 VAL 344 PRO 323 ILE
				No. of residues in known binding	23
				Human similar targets	6
Saquinavir	Known similar target molecule				Protease, HIV-1
	Binding properties	3	1	Superposition type	L
				RMSD	0.81 Å
				Amino acid targets of drug	72 ILE 157 GLY 156 VAL
				No. of residues in known binding	31
				Human similar targets	11
			2	Superposition type	R
				RMSD	0.92 Å
				Amino acid targets of drug	122 VAL 119 PRO 80 ILE
				No. of residues in known binding	31
				Human similar targets	5
			3	Superposition type	L
				RMSD	0.68 Å
				Amino acid targets of drug	321 VAL 344 PRO 323 ILE
				No. of residues in known binding	22
				Human similar targets	9
Tipranavir	Known similar target molecule				Protease, HIV-1
	Binding properties	1	1	Superposition type	L
				RMSD	0.88 Å
				Amino acid targets of drug	72 ILE 157 GLY 156 VAL
				No. of residues in known binding	27
				Human similar targets	3
Torimifene	Known similar target molecule				ENV, Glycoprotein-1, Zaire ebola virus
	Binding properties	1	1	Superposition type	L
				RMSD	0.88 Å
				Amino acid targets of drug	72 ILE 157 GLY 156 VAL
				No. of residues in known binding	27
				Human similar targets	3

Features of different drug binding motifs. *HIV-1* Human Immunodeficiency virus 1, *RMSD* Root Mean Square Deviation, *Å* angstrom, *ILE* isoleucine, *GLY* glycine, *VAL* valine, *LEU* leucine, *PRO* proline, *ASP* aspartic acid

**Table 11:** Possible binding sites of NSP16-NSP10 complex against known anti-viral drugs

Drugs		Total binding sites			(7BQ7) NSP16-NSP10 complex of COVID-19
Amprenavir	Known similar target molecule				Protease, HIV-1
	Binding properties	2	1	Superposition type	L
				RMSD	0.54 Å
				Amino acid targets of drug	106 ASP 70 GLY 71 ALA
				No. of residues in known binding	18
				Human similar targets	4
			2	Superposition type	L
				RMSD	0.96 Å
				Amino acid targets of drug	157 ILE 208 GLY 207 ILE
				No. of residues in known binding	24
				Human similar targets	6
Atazanavir	Known similar target molecule				Protease, HIV-1
	Binding properties	3	1	Superposition type	R
				RMSD	0.94 Å
				Amino acid targets of drug	107 PRO 108 VAL 38 ILE
				No. of residues in known binding	19
				Human similar targets	4
			2	Superposition type	L
				RMSD	0.92 Å
				Amino acid targets of drug	78 ARG 107 PRO 108 VAL
				No. of residues in known binding	23
				Human similar targets	3
			3	Superposition type	L
				RMSD	0.88 Å
				Amino acid targets of drug	97 ASP 107 ALA 108 ASP
				No. of residues in known binding	18
				Human similar targets	3
Darunavir	Known similar target molecule				Protease, HIV-1
	Binding properties	5	1	Superposition type	L
				RMSD	0.54 Å
				Amino acid targets of drug	106 ASP 70 GLY 71 ALA
				No. of residues in known binding	27
				Human similar targets	7
			2	Superposition type	R
				RMSD	0.95 Å
				Amino acid targets of drug	78 ARG 107 PRO 108 VAL
				No. of residues in known binding	21
				Human similar targets	6
			3	Superposition type	R
				RMSD	0.93 Å
				Amino acid targets of drug	26 ALA 22 ASP 21 VAL
				No. of residues in known binding	21
				Human similar targets	6
			4	Superposition type	R
				RMSD	0.87 Å
				Amino acid targets of drug	121 ALA 290 ILE 288 VAL
				No. of residues in known binding	19
				Human similar targets	13
			5	Superposition type	L
				RMSD	1.00 Å
				Amino acid targets of drug	85 LEU 96 VAL 67 VAL
				No. of residues in known binding	22
				Human similar targets	6
Grazoprevir	Known similar target molecule				Protease, HIV-1
	Binding properties	2	1	Superposition type	R
				RMSD	1.02 Å
				Amino acid targets of drug	55 ILE 95 LYS 94 GLY
				No. of residues in known binding	16
				Human similar targets	8
			2	Superposition type	R
				RMSD	1.09 Å
				Amino acid targets of drug	119 HIS 294 VAL 293 ASP
				No. of residues in known binding	17
				Human similar targets	8
Indinavir	Known similar target molecule				Polyprotein, HIV-1
	Binding properties	1	1	Superposition type	L
				RMSD	0.94 Å
				Amino acid targets of drug	78 ARG 107 PRO 108 VAL
				No. of residues in known binding	24
				Human similar targets	2
Lopinavir	Known similar target molecule				Protease, HIV-1
	Binding properties	1	1	Superposition type	L
				RMSD	0.84 Å
				Amino acid targets of drug	78 ARG 107 PRO 108 VAL
				No. of residues in known binding	23
				Human similar targets	6
Nelfinavir	Known similar target molecule				Protease, HIV-1
	Binding properties	1	1	Superposition type	L
				RMSD	0.50 Å
				Amino acid targets of drug	106 ASP 70 GLY 71 ALA
				No. of residues in known binding	30
				Human similar targets	8
Rimantadine	Known similar target molecule				M2 protein, Influeza A
	Binding properties	2	1	Superposition type	R
				RMSD	0.86 Å
				Amino acid targets of drug	197 VAL 199 ALA 200 SER
				No. of residues in known binding	10
				Human similar targets	0
			2	Superposition type	L
				RMSD	0.93 Å
				Amino acid targets of drug	32 ALA 33 SER 34 GLY
				No. of residues in known binding	9
				Human similar targets	0
Ritonavir	Known similar target molecule				Protease, HIV-1
	Binding properties	2	1	Superposition type	L
				RMSD	0.77 Å
				Amino acid targets of drug	97 ASP 107 ALA 108 ASP
				No. of residues in known binding	18
				Human similar targets	4
			2	Superposition type	L
				RMSD	0.98 Å
				Amino acid targets of drug	78 ARG 107 PRO 108 VAL
				No. of residues in known binding	23
				Human similar targets	4
Saquinavir	Known similar target molecule				Protease, HIV-1
	Binding properties	4	1	Superposition type	L
				RMSD	1.02 Å
				Amino acid targets of drug	157 ILE 208 GLY 207 ILE
				No. of residues in known binding	29
				Human similar targets	7
			2	Superposition type	R
				RMSD	1.01 Å
				Amino acid targets of drug	257 THR 62 PRO 61 VAL
				No. of residues in known binding	22
				Human similar targets	4
			3	Superposition type	L
				RMSD	0.80 Å
				Amino acid targets of drug	78 ARG 107 PRO 108 VAL
				No. of residues in known binding	31
				Human similar targets	8
			4	Superposition type	L
				RMSD	0.52 Å
				Amino acid targets of drug	106 ASP 70 GLY 71 ALA
				No. of residues in known binding	31
				Human similar targets	7
Tipranavir	Known similar target molecule				Protease, HIV-1
	Binding properties	1	1	Superposition type	L
				RMSD	0.53 Å
				Amino acid targets of drug	106 ASP 70 GLY 71 ALA
				No. of residues in known binding	27
				Human similar targets	7

Features of different drug binding motifs. *HIV-1* Human Immunodeficiency virus 1, *RMSD* Root Mean Square Deviation, *Å* angstrom, *ILE* isoleucine, *GLY* glycine, *VAL* valine, *LEU* leucine, *PRO* proline, *ASP* aspartic acid

**Table 12:** Comparison of drug binding motifs of analyzed NSPs for antiviral drugs

	7K3N (Nsp1)	6WEY (Nsp3)	6M03 (Nsp5)	7JLTNsp7-8	6W4B (Nsp9)	6ZCTNsp10	6M71 (Nsp7/8/12)	7NIO Nsp13	5C8S (Nsp14)	6VWW (Nsp15)	7BQ7 Nsp16-10	Total binding sites
Amphetamine			+									1
Amprenavir	+ + +	+ + + +		+ +			+ + +	+ + +	+ + +	+ +	+ +	**22**
Atazanavir	+					+ +	+	+	+	+	+ + +	10
Darunavir	+ + + + + + + + + +	+ + + + + +	+ +	+		+ + +	+ + + +	+ +	+ + + + + + +	+ + + + +	+ + + + +	**45**
Grazoprevir					+	+ +			+		+ +	6
Indinavir	+		+			+	+	+ + +	+	+ + +	+	*12*
Lopinavir						+		+ +	+ +	+ + +	+	9
Nelfinavir	+ +		+	+			+ +	+			+	8
Nevirapine			+									1
Ribavirin			+		+							2
Rimantadine	+	+ +	+ + +	+	+ + +		+	+ +	+ + + + +		+ +	**20**
Ritonavir			+			+		+			+ +	5
Saquinavir	+ +	+		+		+	+ + +	+	+ +	+ + +	+ + + +	**18**
Tipranavir	+	+ +	+		+		+ +	+	+ +	+	+	*12*

Among fourteen drugs four (Amprenavir, Darunavir, Rimantadine, Saquinavir) have very significant and the other two (Indinavir, Tipranavir) have moderatenumber of binding motifs. ‘ + ’ sign indicates no. of drug binding motifs

**Table 13:**
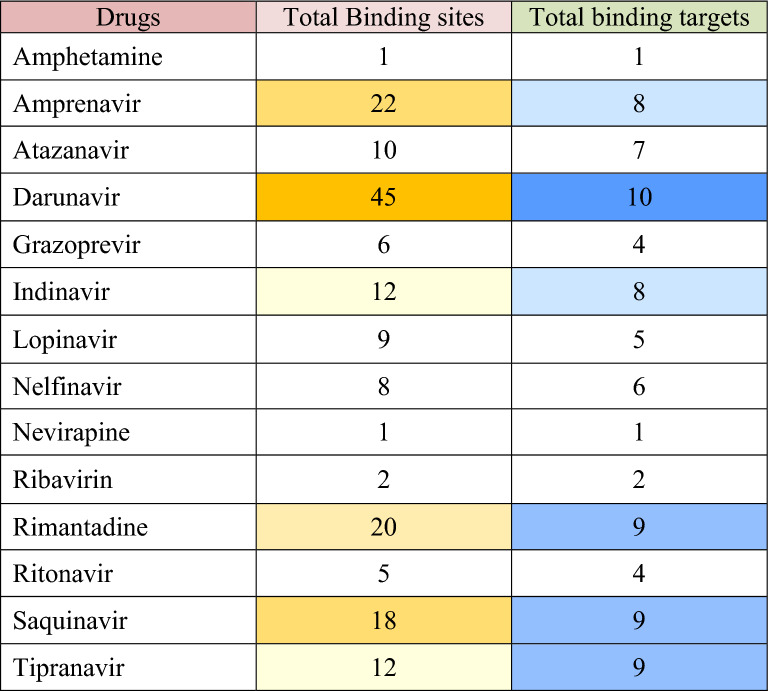
Comparison of NSPs binding of the drugs analyzed

Coloured boxes indicate significant binding properties

**Fig. 1:**
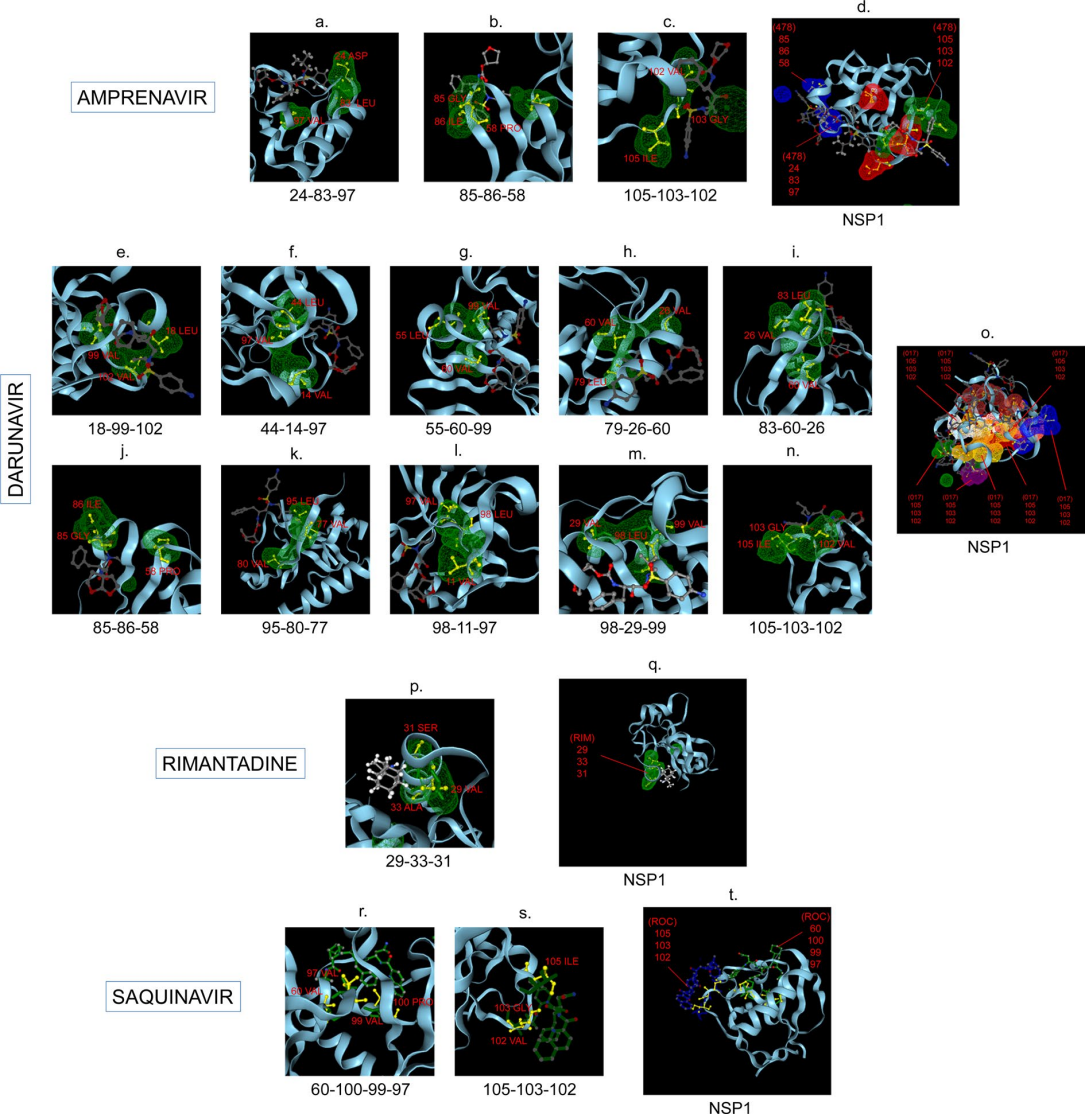
3D-binding interfaces of NSP1with Amprenavir, Darunavir, Rimantadine &Saquinavir.** a**–**c** Binding motifs of Amprenavir. **d** All the binding motifs of Amprenavir. **e**–**n **Binding interfaces of Darunavir. **o** All the binding motifs of Darunavir. **p**, **q** Rimantadine binding motif. **r**, **s** Saquinavir binding motifs and **t** All the motifs on NSP1. Numbers indicate the motif forming amino acids. Three letter codes of amino acids have been mentioned

**Fig. 2:**
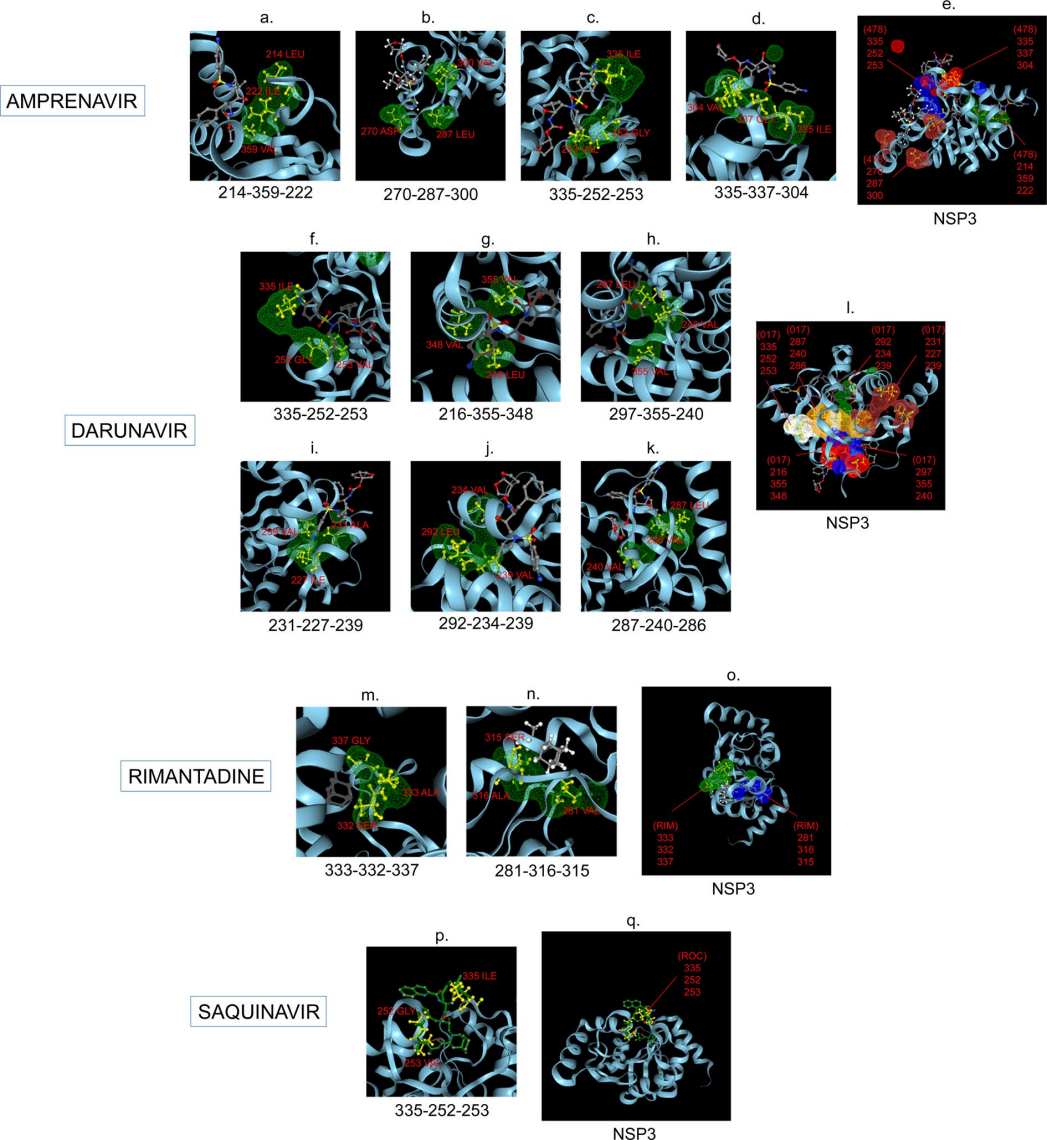
3D-binding interfaces of NSP3 with Amprenavir, Darunavir, Rimantadine &Saquinavir. **a**–**d** Binding motifs of Amprenavir. **e** All the binding motifs of Amprenavir together. **f**–**k **Binding interfaces of Darunavir. **L **Combined binding motifs of Darunavir. **m**, **n** Rimantadine binding motifs. **o** All motifs of RIM. **p**, **q** Saquinavir binding motif. Numbers indicate the motif forming amino acids. Three letter codes of amino acids have been mentioned

**Fig. 3:**
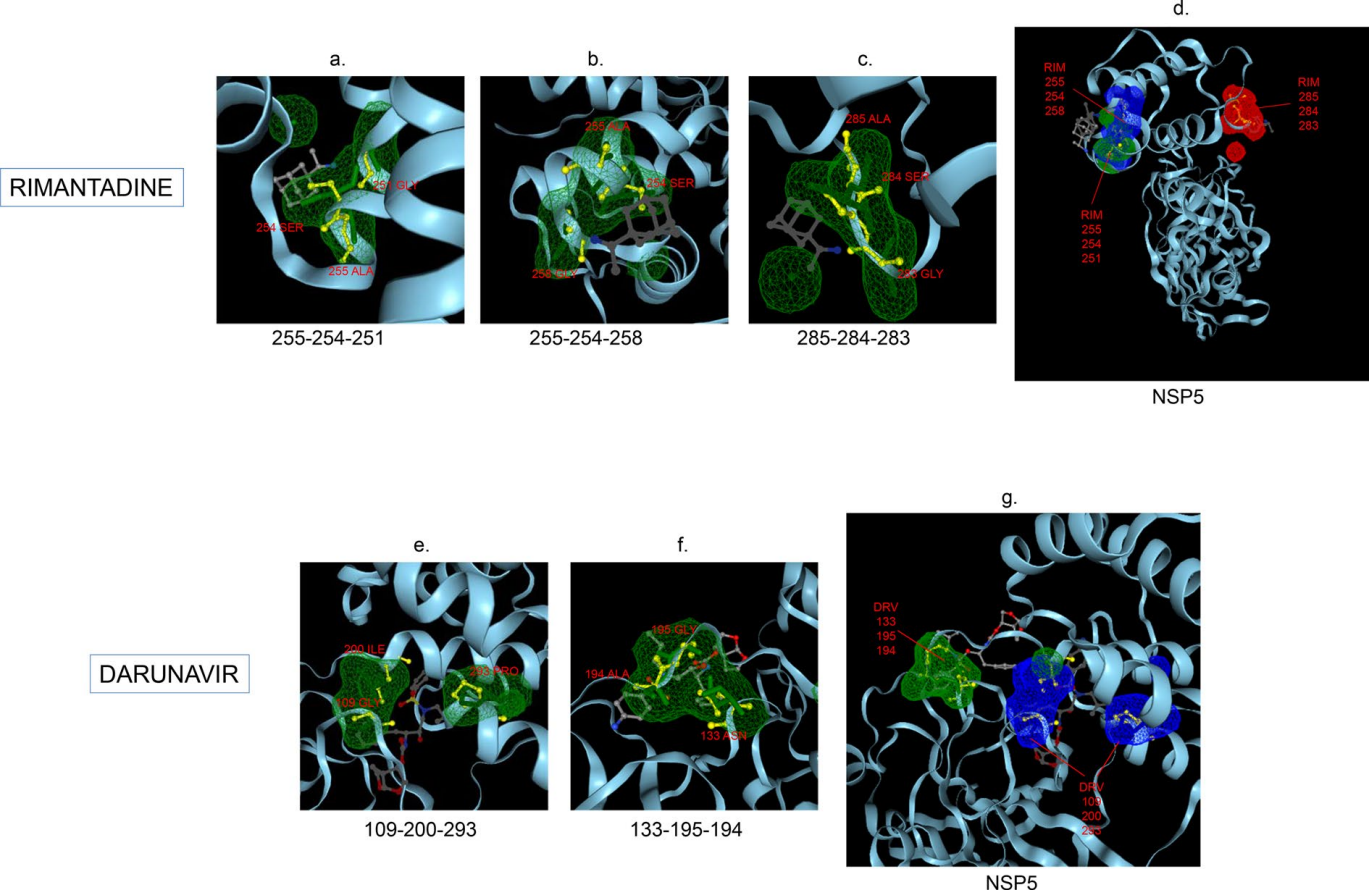
3D-binding interfaces of NSP5 with Darunavir&Rimantadine.** a**–**c** Binding motifs of Rimantadine. **d** All the binding motifs of RIM on NSP5. **e**, **f** Binding interfaces of Darunavir. **g** All the binding motifs of Darunavir. Numbers indicate the motif forming amino acids. Three letter codes of amino acids have been mentioned

**Fig. 4:**
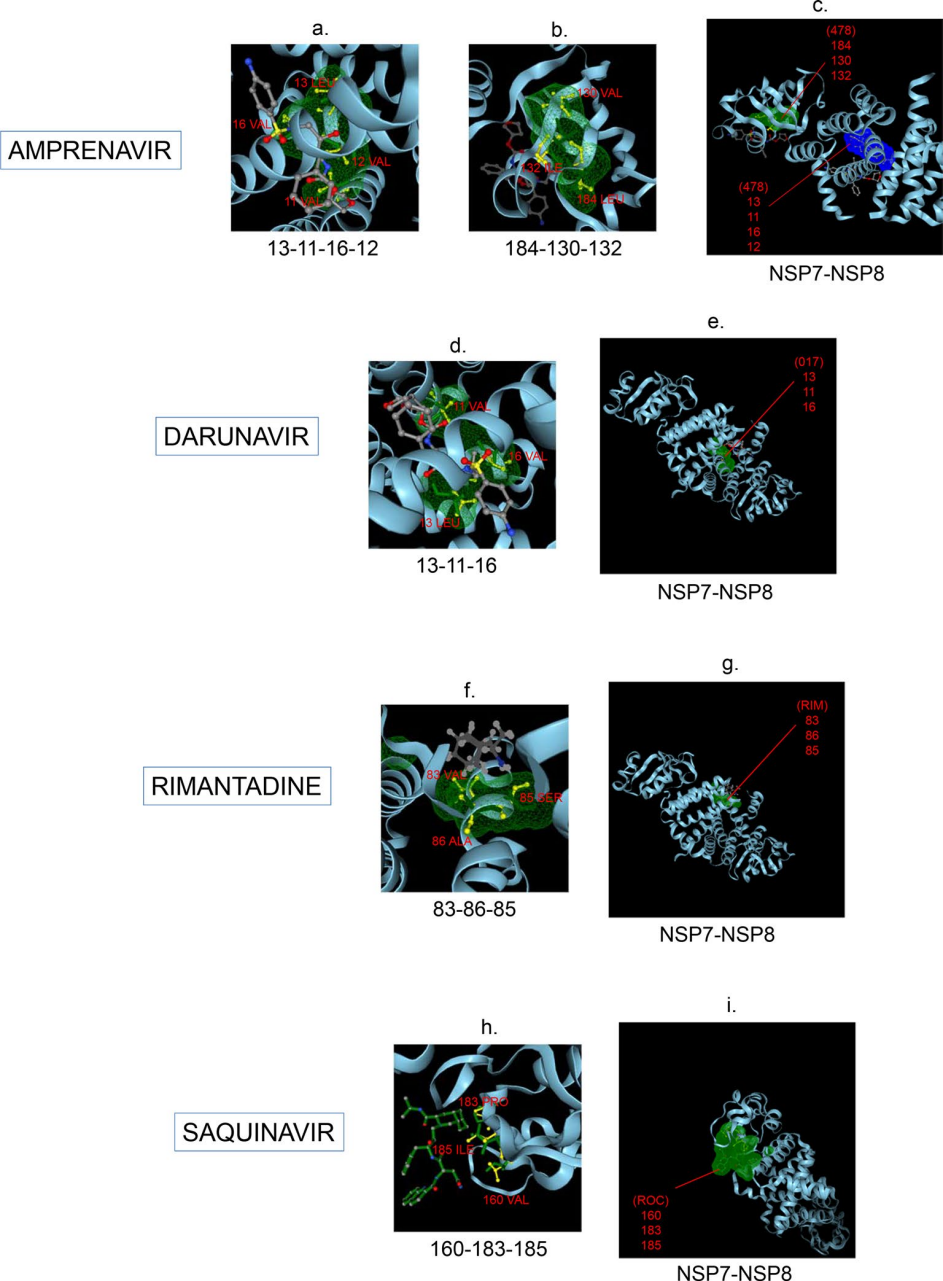
3D-binding interfaces of NSP7-8 complex with Amprenavir, Darunavir, Rimantadine &Saquinavir.** a**, **b.** Binding motifs of Amprenavir. **c** All the binding motifs of Amprenavir together. **d**, **e **Binding interfaces of Darunavir. **f**, **g** Rimantadine binding motifs. **h**, **i** Saquinavir binding motif. Numbers indicate the motif forming amino acids. Three letter codes of amino acids have been mentioned

**Fig. 5:**
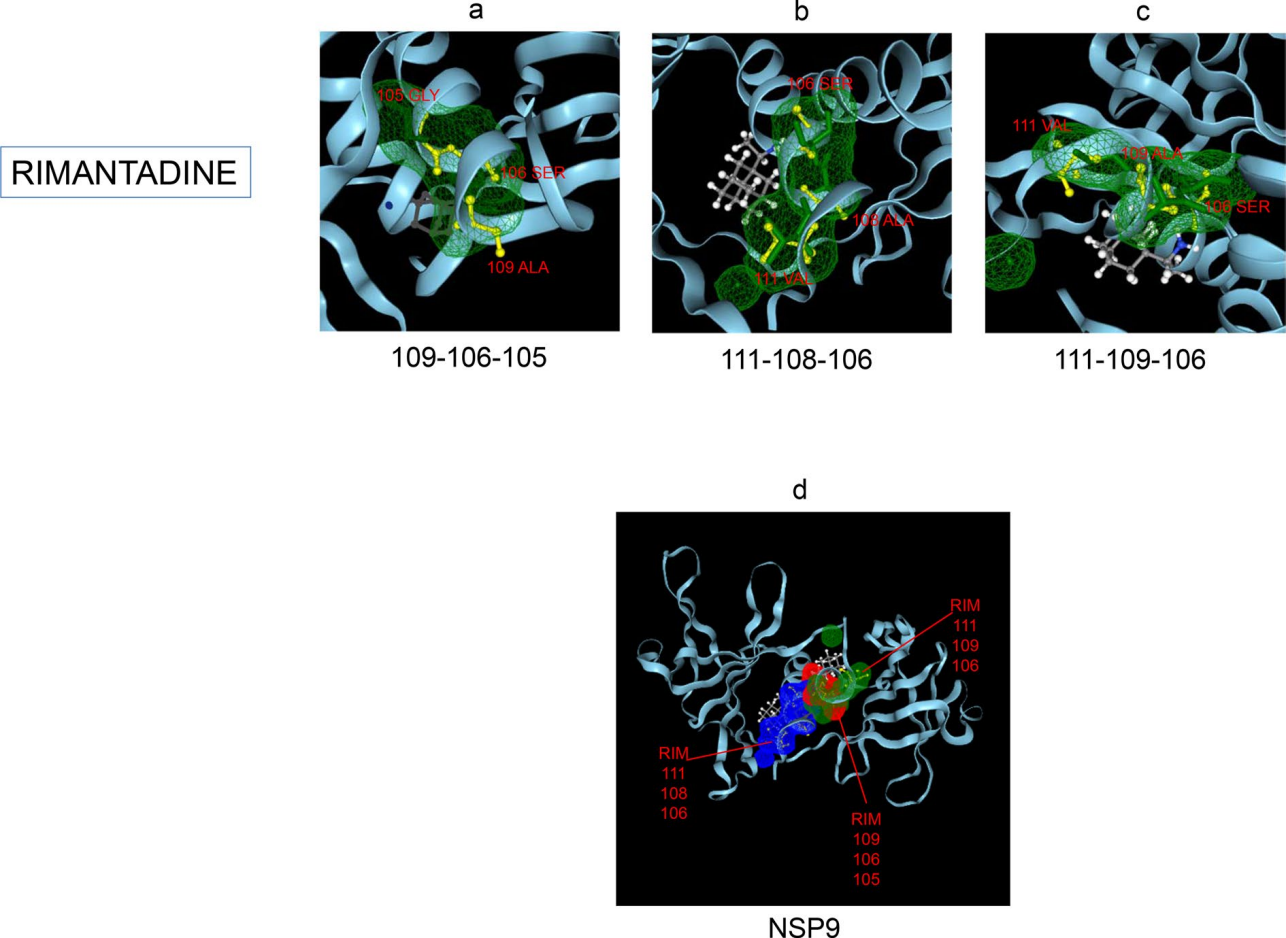
3D-binding interfaces of NSP9 with Rimantadine.** a**–**c **Three binding motifs of Rimantadine.** d** All the binding motifs of RIM together. Numbers indicate the motif forming amino acids. Three letter codes of amino acids have been mentioned

**Fig. 6:**
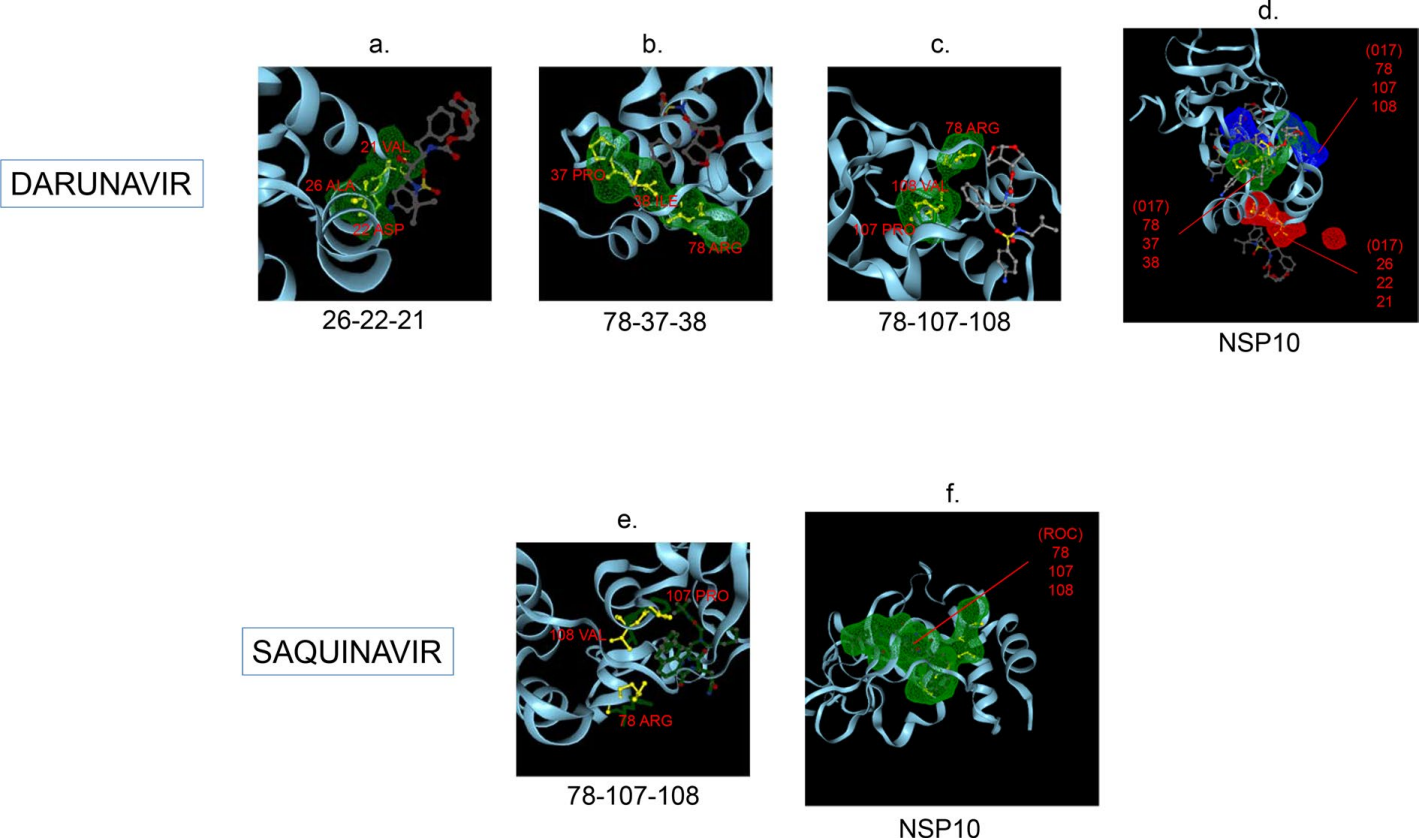
3D-binding interfaces of NSP10with Darunavir&Saquinavir.** a**–**c.** Binding motifs of Darunavir. **d** Combined binding motifs of Darunavir. **e**, **f** Saquinavir binding motif. Numbers indicate the motif forming amino acids. Three letter codes of amino acids have been mentioned

**Fig. 7:**
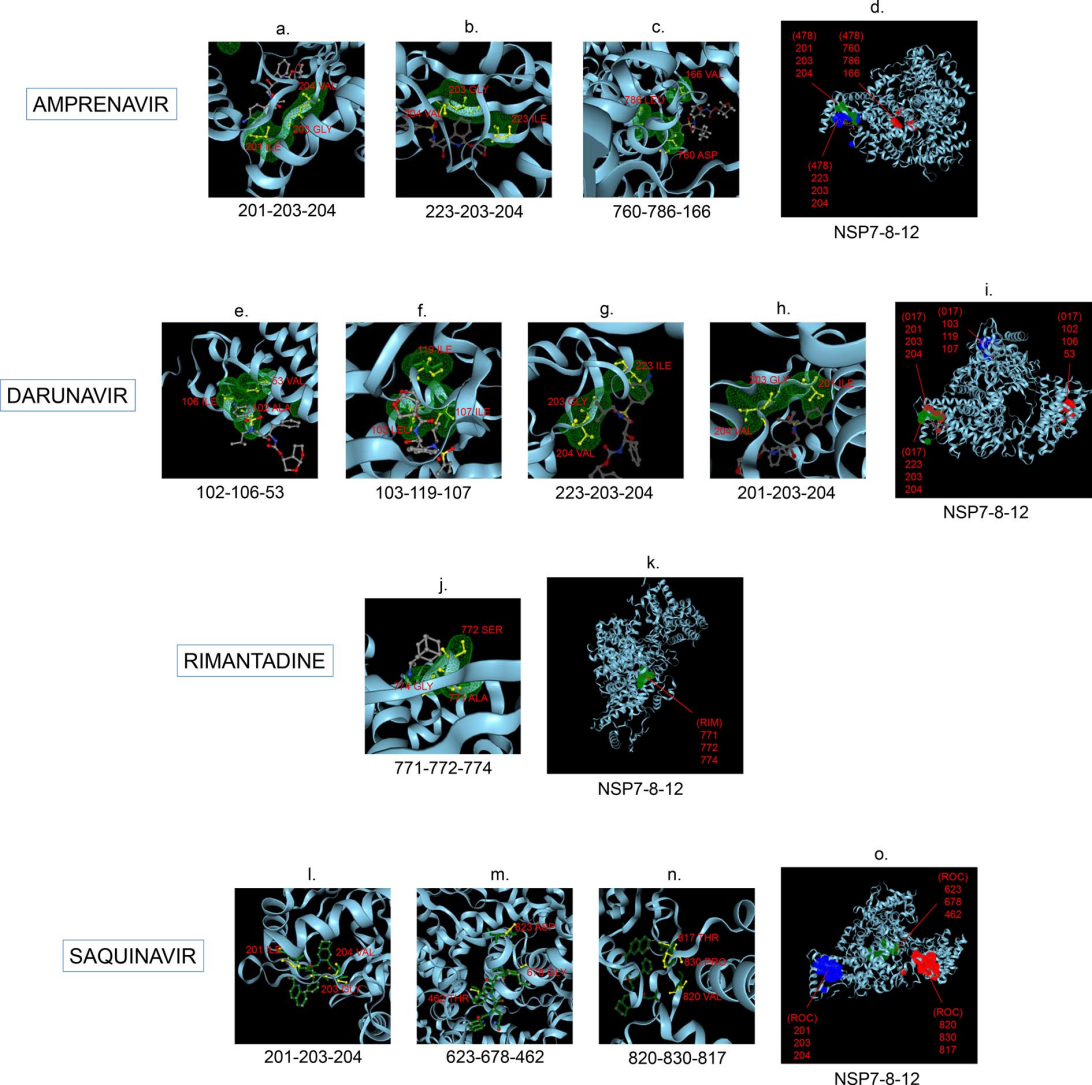
3D-binding interfaces of NSP7-8–12 complex with Amprenavir, Darunavir, Rimantadine &Saquinavir. **a**–**c** Binding motifs of Amprenavir. **d** All the binding motifs of Amprenavir together. **e**–**h **Binding interfaces of Darunavir. **i** Combined binding motifs of Darunavir. **j**, **k** Rimantadine binding motifs. **l**–**n** Saquinavir binding motifs. **o** Combined motifs of ROC. Numbers indicate the motif forming amino acids. Three letter codes of amino acids have been mentioned

**Fig. 8:**
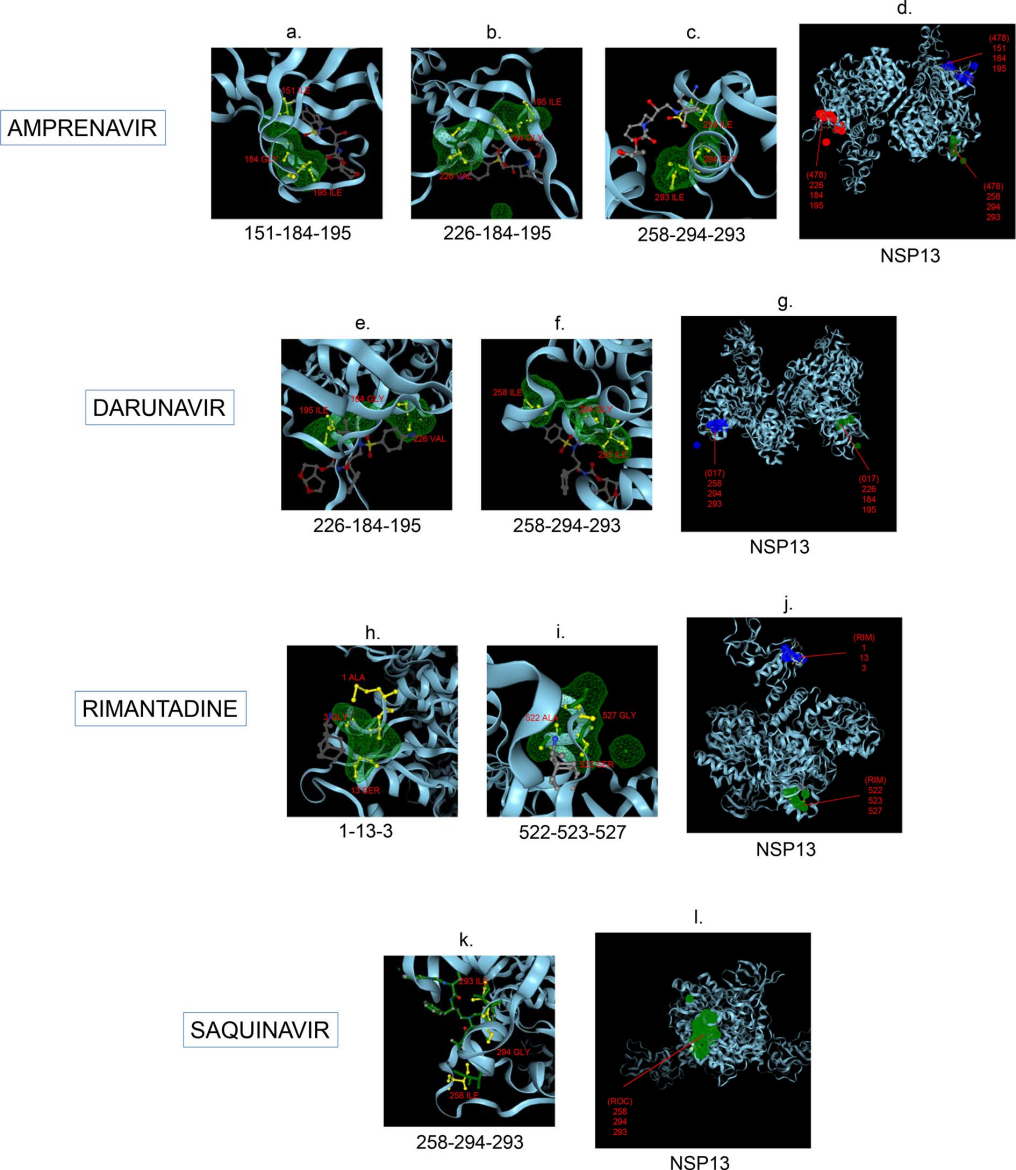
3D-binding interfaces of NSP13 with Amprenavir, Darunavir, Rimantadine &Saquinavir.** a–c** Binding motifs of Amprenavir. **d** All the binding motifs of Amprenavir together. **e**, **f.** Binding interfaces of Darunavir. **g** Combined binding motifs of Darunavir. **h**, **i.** Rimantadine binding motifs. **j** All motifs of RIM. **k**, **l** Saquinavir binding motif. Numbers indicate the motif forming amino acids. Three letter codes of amino acids have been mentioned

**Fig. 9:**
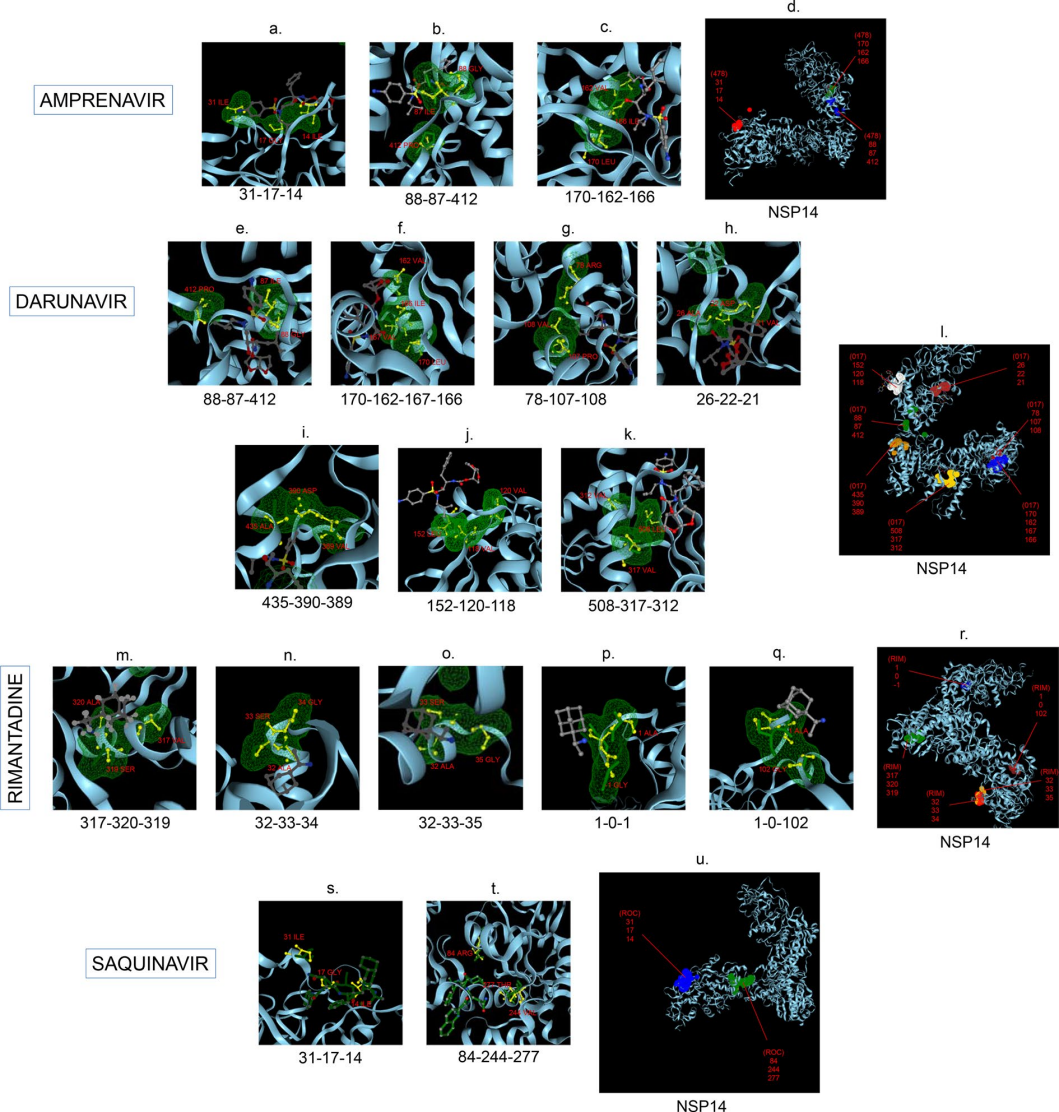
3D-binding interfaces of NSP14 with Amprenavir, Darunavir, Rimantadine &Saquinavir.** a**–**c** Binding motifs of Amprenavir. **d** All the binding motifs of Amprenavir together. **e**–**k.**Binding interfaces of Darunavir. **l** Combined binding motifs of Darunavir. **m**–**q** Rimantadine binding motifs. **r** All motifs of RIM. **s, t** Saquinavir binding motifs. **u** All the Saquinavir motifs together. Numbers indicate the motif forming amino acids. Three letter codes of amino acids have been mentioned

**Fig. 10:**
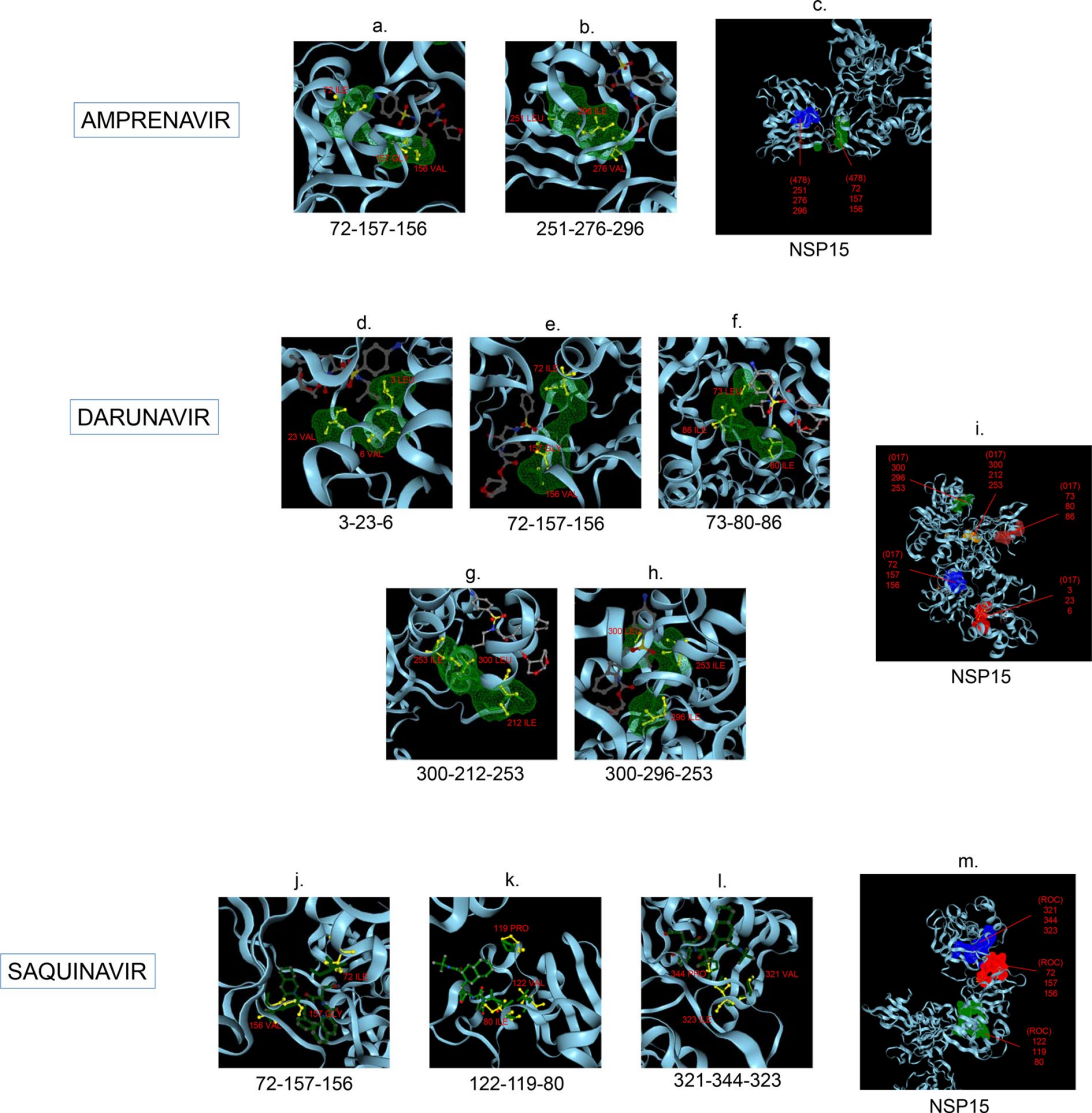
3D-binding interfaces of NSP15 with Amprenavir, Darunavir&Saquinavir. **a**, **b** Binding motifs of Amprenavir. **c** All the binding motifs of Amprenavir together. **d**–**h **Binding interfaces of Darunavir. **i** Combined binding motifs of Darunavir. **j**–**l** Saquinavir binding motifs. **m** All the ROC binding interfaces. Numbers indicate the motif forming amino acids. Three letter codes of amino acids have been mentioned

**Fig. 11:**
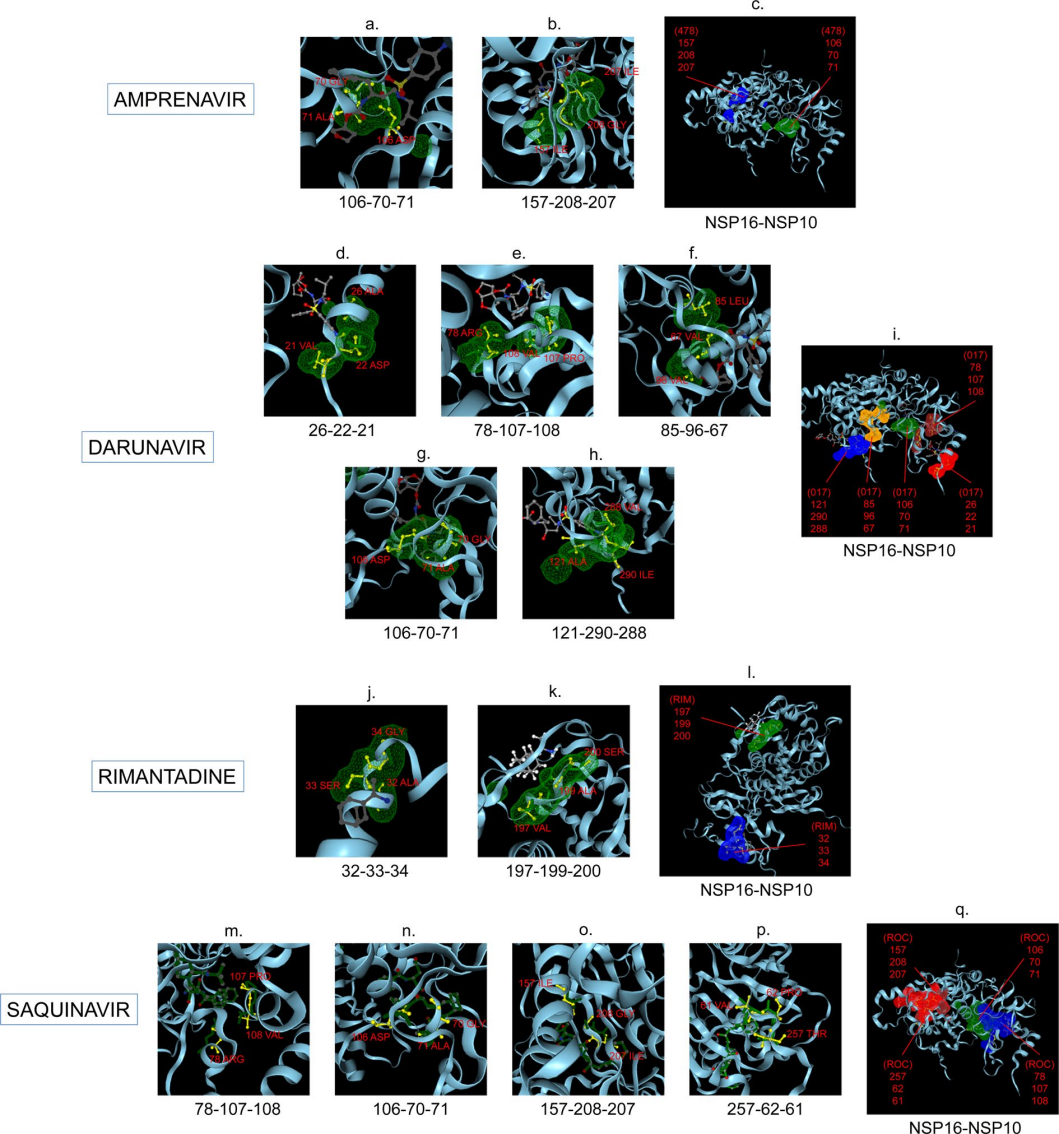
3D-binding interfaces of NSP16-10 complex with Amprenavir, Darunavir, Rimantadine &Saquinavir. **a**, **b** Binding motifs of Amprenavir. **c** All the binding motifs of Amprenavir together. **d**–**h **Binding interfaces of Darunavir. **i** Combined binding motifs of Darunavir. **j**, **k** Rimantadine binding motifs. **l** All motifs of RIM. **m**–**p** Saquinavir binding motifs. **q** All the Saquinavir binding interfaces. Numbers indicate the motif forming amino acids. Three letter codes of amino acids have been mentioned

Amphetamine (DB00182) targeted only a single binding interface on Nsp5 (6M03) (Tables 3, 12, 13). Amprenavir (DB00701) targeted four binding motifs on Nsp3 (6WEY), three motifs onNsp1 (7K3N), Nsp7-8-12 complex (6M71), Nsp13 (7NIO) and Nsp14 (5C8S), and two binding motifs on Nsp7-8 complex (7JLT), Nsp15 (6VWW) and Nsp16-10 complex (7BQ7) (Tables 2, 1, 7, 8, 9, 4, 10, 11, 12, Figs. 1, 2,3, 4, 5, 6, 7, 8, 9, 10 and 11). Atazanavir (DB01072) targeted three motifs on Nsp16-10 complex (7BQ7), two motifs on Nsp10 (6ZCT) and single motif each on Nsp1, Nsp7-8-12, Nsp13, Nsp14 and Nsp15 (Tables 11, 6, 12). Darunavir (DB01264) is the most promising drug as it targeted the greatest number of binding motifs and targeted every molecule except Nsp9. It targeted ten motifs on Nsp1 (7K3N), seven motifs on Nsp14 (5C8S), six motifs on Nsp3 (6WEY), five motifs on Nsp15 (6VWW) and Nsp16-10 complex (7BQ7), four motifs on Nsp7-8-12 complex (6M71), three motifs on Nsp10 (6ZCT), two motifs each on Nsp5 (6M03) and Nsp13 (7NIO), respectively and a single motif on Nsp7-8 complex (Tables 1, 9, 2, 10, 11, 7, 6, 3, 8, 4, 12, Figs. 1, 2, 3, 4, 5, 6, 7, 8, 9, 10 and 11). Grazoprevir (DB11575) targeted two motifs, on Nsp10 (6ZCT) and two on Nsp16-10 complex (7BQ7) and single motif each on Nsp9 and Nsp14 (Tables 6, 11, 5, 9, 12). Indinavir (DB00224) significantly targeted three motifs, each on Nsp13 (7NIO) and Nsp15 (6VWW) (Tables 8, 10, 12). Lopinavir significantly targeted three motifs on Nsp15 and 2 motifs each on Nsp13 and Nsp14 (Tables 10, 8, 9). Nelfinavir targeted two interfaces on Nsp1 and Nsp7-8–12 complexes (Tables 1, 7). On the other hand, Nevirapine targeted only a single motif on Nsp5 (Table 3). Rimantadine (DB00478) significantly targeted five binding interfaces on Nsp14 (5C8S), three binding motifs each on Nsp5 (6M03) and Nsp9 (6W4B), and two motifs on Nsp3 (6WEY), Nsp13 (7NIO), Nsp16-10 (7BQ7) and a single motif on Nsp1, Nsp7-8 and Nsp7-8-12 complex (Tables 9, 3, 5, 2, 8, 11, 1, 4, 7, 12, Figs. 1, 2, 3, 4, 5, 6, 7, 8, 9, 10 and 11). Ritonavir targeted two motifs on Nsp16-10 complex (Table 11). Saquinavir (DB01232) targeted four motifs on Nsp16-10 complex (7BQ7), three interfaces each on Nsp7-8–12 (6M71) and Nsp15 (6VWW), two motifs on Nsp1 and Nsp14 (5C8S) and a single motif on Nsp3, Nsp7-8, Nsp10 and Nsp13 (Tables 11, 7, 10, 1, 9, 3, 4, 6, 8, Figs. 1, 2, 3, 4, 5, 6, 7, 8, 9, 10 and 11). Finally, Tipranavir (DB00932) targeted two binding motifs; each on Nsp3, Nsp7-8–12 complex and Nsp14 (Tables 3, 7, 9), whereas single binding interface each on Nsp1, Nsp5, Nsp9, Nsp13, Nsp15 and Nsp16-10 (Table 12).

All the binding results were further compiled and analyzed. Results revealed that Darunavir (DB01264) had 45 unique binding sites and targeted 10 SARS-CoV-2 PDB entries or 10 NSPs (Tables 12, 13). The Lowest Root Mean Square Deviation (RMSD) value of Darunavir among all the target molecules was 0.54 Å for Nsp16-10 complex and maximum number of residues involved in interaction was 27 (Tables 1, 2, 3, 4, 5, 6, 7, 8, 9, 10 and 11). Significant binding interfaces were again targeted by Amprenavir (DB00701) and Saquinavir (DB01232) with 22 and 18 (Tables 12, 13), respectively. The two drugs had eight and nine binding partners, respectively (Tables 12, 13). The lowest RMSDs for them were 0.54 Å and 0.52 Å and maximum residues involved in drug-target binding were 28 and 31, respectively (Tables 1, 2, 3, 4, 5, 6, 7, 8, 9, 10 and 11). Additionally, Rimantadine (DB00478) had 20 drug binding motifs that targeted nine binding partners (Tables 12, 13) with the lowest RMSD value of 0.67 Å and maximum number of residues involved in binding were 10 (Tables 1, 2, 3, 4, 5, 6, 7, 8, 9, 10 and 11). Again, Tipranavir (DB00932) and Indinavir (DB00224) both showed 12 binding motifs for nine and eight binding partners, respectively (Tables 12, 13). Lowest RMSD values for these two drugs were 0.53 Å and 0.72 Å and maximum number of residues involved in binding were 27 and 24, respectively (Tables 1, 2, 3, 4, 5, 6, 7, 8, 9, 10 and 11).

Results showed that Darunavir, Amprenavir, Rimantadine, Saquinavir, Tipranavir and Indinavir were more effective in targeting the twelve SARS-CoV-2 proteins and their complexes (Tables 12, 13). Darunavir is a nonpeptidic benzenesulfonamide inhibitor that targets active site of HIV-1 protease [38, 39]. Amprenavir is a hydroxyethylamine sulfonamide derivative that inhibits HIV-1 protease [40, 41]. Rimantadine is an alkylamine that specifically targets Influenza A virus M2 protein [42–44]. Saquinavir is a L-asparagine derivative that acts as HIV-1 protease inhibitor [45, 46]. Tipranavir is a sulfonamide that acts as HIV-1 protease inhibitor [47]. Moreover, Indinavir is a piperazinecarboxamide having HIV-1 protease inhibitory activity [48, 49]. The drug binding interfaces determined in the present study is very much significant as the analysis considered previously known potent binding information between specific drugs and target proteins that were again supported by very low RMSD values of the motifs such as 0.54 Å for both Darunavir and Amprenavir, 0.52 Å for Saquinavir and 0.67 Å for Rimantadine (Tables 1, 2, 3, 4, 5, 6, 7, 8, 9, 10 and 11). RMSD values well below 1.0 was indicative of presence of similar drug binding structures or motifs as in active site of HIV-1 protease or M2 of Influenza A and these results emphasized that the selected drugs would effectively target those similar interfaces found on different NSPs of SARS-Cov2 to inhibit them. Furthermore, considering the produced results, it has been proposed that combination of Darunavir, Amprenavir and Rimantadine could effectively target and inhibit all the NSPs that were studied. Darunavir targeted all NSPs except Nsp9, whereas Amprenavir targeted all except Nsp5, Nsp9 and Nsp10 and interestingly Rimantadine complementarily and significantly targeted Nsp5 and Nsp9, which are two key enzymes (Tables 12, 13). However, it has been reported that Darunavir was unable to protect HIV patients from SARS-Cov2 infection who were under Darunavir treatment [50]. Though, the claim has to be experimentally proven. In such cases, if Darunavir fails to prevent infection, then another potent inhibitor Saquinavir, having similar target profiles, could be used in combination along with Amprenavir and Rimantadine, in replacement of Darunavir (Tables 12, 13, 14).

**Table 14:** Active site residues of the analyzed SARS-CoV2 enzymes and the inhibitory drug binding motifs

Enzymes of COVID-19	GASS-WEB predicted Active site residues	Template PDB ID	Avg. fitness & template resolution	Drug binding enzyme residues			
				Amprenavir	Darunavir	Rimantadine	Saquinavir
NSP3-6WEY	CYS 285 ALA 264 ARG 352 HIS 295 CYS 296 VAL 253 LYS 376 ASP 366 LYS 367 LYS 362 HIS 290 LYS 215 VAL 355 VAL 228 ASP 339	IN2C Nitrogenase complex from *Azotobacter vinelandii*	288, 3.00	335 ILE *252 GLY* *253 VAL* *335 ILE* 337 GLY *304 VAL* *270 ASP* *287 LEU* *300 VAL* 214 LEU 359 VAL 222 ILE	*335 ILE* *252 GLY* *253 VAL* 216 LEU 355 VAL 348 VAL *297 LEU* 240 VAL 231 ALA *227 ILE* 239 VAL 292 LEU 234 VAL *287 LEU* *286 VAL*	*333 ALA* 332 SER *337 GLY* *281 VAL* 316 ALA *315 SER*	*335 ILE* *252 GLY* *253 VAL*
NSP5-6M03	GLU 14 ARG 298 TRP 207 GLN 127 PHE 291 ASP 289 CYS 265 HIS 246 TYR 239 PHE 3 PHE 8 CYS 300 GLU 166 ARG 4 PHE 112 ARG 105 GLN 110 ASP 295	2SQC, Squalene-hopene cyclise of *Alicyclobacillus acidocaldarius*	625, 2.00		*109 GLY* *200 ILE* *293 PRO* *133 ASN* *195 GLY* *194 ALA*	*255 ALA* *254 SER* *251 GLY* 258 GLY 285 ALA 284 SER 283 GLY	
NSP9-6W4B	LYS 87 SER 6 ILE 92 GLY 101 GLY 105 SER 106 SER 47 SER 24	1MT5, Fatty-acid amide hydrolase of *Rattus norvegicus*	152, 2.80			*109 ALA* *106 SER* *105 GLY* *111 VAL* *108 VAL*	
NSP12-6M71	GLU 796 GLU 136 ARG 132 TRP 617 GLN 789 TRP 598 PHE 812 ASP 618 CYS 813 ASP 761 HIS 816 TRP 800 TYR 606 PHE 753 PHE 782 GLU 474 GLN 698 ASP 760 HIS 810 PHE 694 GLN 468 GLU 167 ARG 349 TRP 162 TRP 290 PHE 45 ASP 208 CYS 464 ASP 465 HIS 309 TYR 732 PHE 165 PHE 134 ARG 185	2SQC, Squalene-hopene cyclise of *Alicyclobacillus acidocaldarius*	602, 2.00	223 ILE *203 GLY* *204 VAL* *201 ILE* *760 ASP* 786 LEU *166 VAL*	223 ILE *203 GLY* *204 VAL* *201 ILE* 103 LEU *119 ILE* 107 ILE 102 ALA *106 ILE* *53 VAL*	*771 ALA* *772 SER* *774 GLY*	*820 VAL* 830 PRO *817 THR* *623 ASP* 678 GLY 462 THR *201 ILE* *203 GLY* *204 VAL*
NSP13-7NIO	GLU 418 GLU 420 ARG 427 TRP 114 GLN 281 PHE 475 ASP 580 CYS 556 ASP 578 HIS 554 TYR 515 PHE 422 PHE 561 GLU 375 ASP 534 HIS 482 TRP 167 ARG 560 GLU 551 GLU 498 TRP 506 GLN 492 ASP 583 TRP 167 PHE 546 GLN 518 PHE 511 HIS 554 TYR 120 PHE 587	2SQC, Squalene-hopene cyclise of *Alicyclobacillus acidocaldarius*	763, 2.00	*258 ILE* 294 GLY 293 ILE *151 ILE* 184 GLY *195 ILE* *226 VAL* 184 GLY	*258 ILE* 294 GLY 293 ILE *226 VAL* 184 GLY *195 ILE*	*01 ALA* 13 SER *03 GLY* 522 ALA *523 SER* *527 GLY*	*258 ILE* 294 GLY 293 ILE
NSP14-5C8S	GLU 365 GLU 364 ARG 310 TRP 348 GLN 354 TRP 385 PHE 384 ASP 352 CYS 382 ASP 432 HIS 330 TRP 292 TYR 368 PHE 367 PHE 377 PHE 350 ASP 375 ARG 289 GLU 302 GLU 284 ARG 278 GLN 259 PHE 286 CYS 356 HIS 424 TYR 420 PHE 426 CYS 382 ASP 291 CYS 356	2SQC, Squalene-hopene cyclise of *Alicyclobacillus acidocaldarius*	585, 2.00	88 GLY *87 ILE* *412 PRO* 170 LEU 162 VAL 166 ILE *31 ILE* 17 GLY *14 ILE*	88 GLY 87 ILE 412 PRO 170 LEU 162 VAL 167 VAL 166 ILE *78 ARG* 107 PRO *108 VAL* *26 ALA* 22 ASP 21 VAL 435 ALA *390 ASP* *389 VAL* *152 LEU* *120 VAL* *118 VAL* 508 LEU 317 VAL 312 VAL	317 VAL 320 ALA 319 SER *32 ALA* *33 SER* *34 GLY* *35 GLY*	*31 ILE* 17 GLY *14 ILE* *84 ARG* 244 VAL 277 THR
NSP15-6VWW	GLU 69 GLU 146 ARG 127 TRP 87 GLN 160 PHE 44 ASP 88 CYS 103 ASP 92 HIS 15 TYR 89 PHE 123 TRP 59 PHE 56 PHE 177 GLU 69 GLU 22 GLU 42 ARG 62 GLN 19 ASP 107 HIS 96 PHE 16 PHE 44 GLU 4 GLN 19	2SQC, Squalene-hopene cyclise of *Alicyclobacillus acidocaldarius*	645, 2.00	72 ILE 157 GLY *156 VAL* 251 LEU *276 VAL* 296 ILE	*3 LEU* *23 VAL* 6 VAL 72 ILE 157 GLY *156 VAL* 73 LEU *80 ILE* *86 ILE* 300 LEU *212 ILE* 253 ILE 296 ILE 253 ILE		72 ILE 157 GLY *156 VAL* 122 VAL *119 PRO* *80 ILE* 321 VAL 344 PRO 323 ILE
NSP16-7BQ7	GLU 217 ARG 216 TRP 88 GLN 158 GLU 147 TRP 189 PHE 205 ASP 125 CYS 51 ASP 130 HIS 69 TRP 124 TYR 47 PHE 156 PHE 187 ASP 97 TRP 190 PHE 70 GLU 173 GLU 23 ARG 232 GLN 3 PHE 193 PHE 150 TRP 231 GLN 6 PHE 149	2SQC, Squalene-hopene cyclise of *Alicyclobacillus acidocaldarius*	475, 2.00	106 ASP *70 GLY* *71 ALA* 157 ILE 208 GLY 207 ILE	106 ASP *70 GLY* *71 ALA* 78 ARG 107 PRO 108 VAL *26 ALA* *22 ASP* *21 VAL* *121 ALA* *290 ILE* 288 VAL 85 LEU 96 VAL 67 VAL	*197 VAL* *199 ALA* *200 SER* *32 ALA* *33 SER* *34 GLY*	157 ILE 208 GLY 207 ILE 257 THR 62 PRO 61 VAL 78 ARG 107 PRO 108 VAL 106 ASP *70 GLY* *71 ALA*

Italic residues were in close proximity with the active sites

**Fig. 12:**
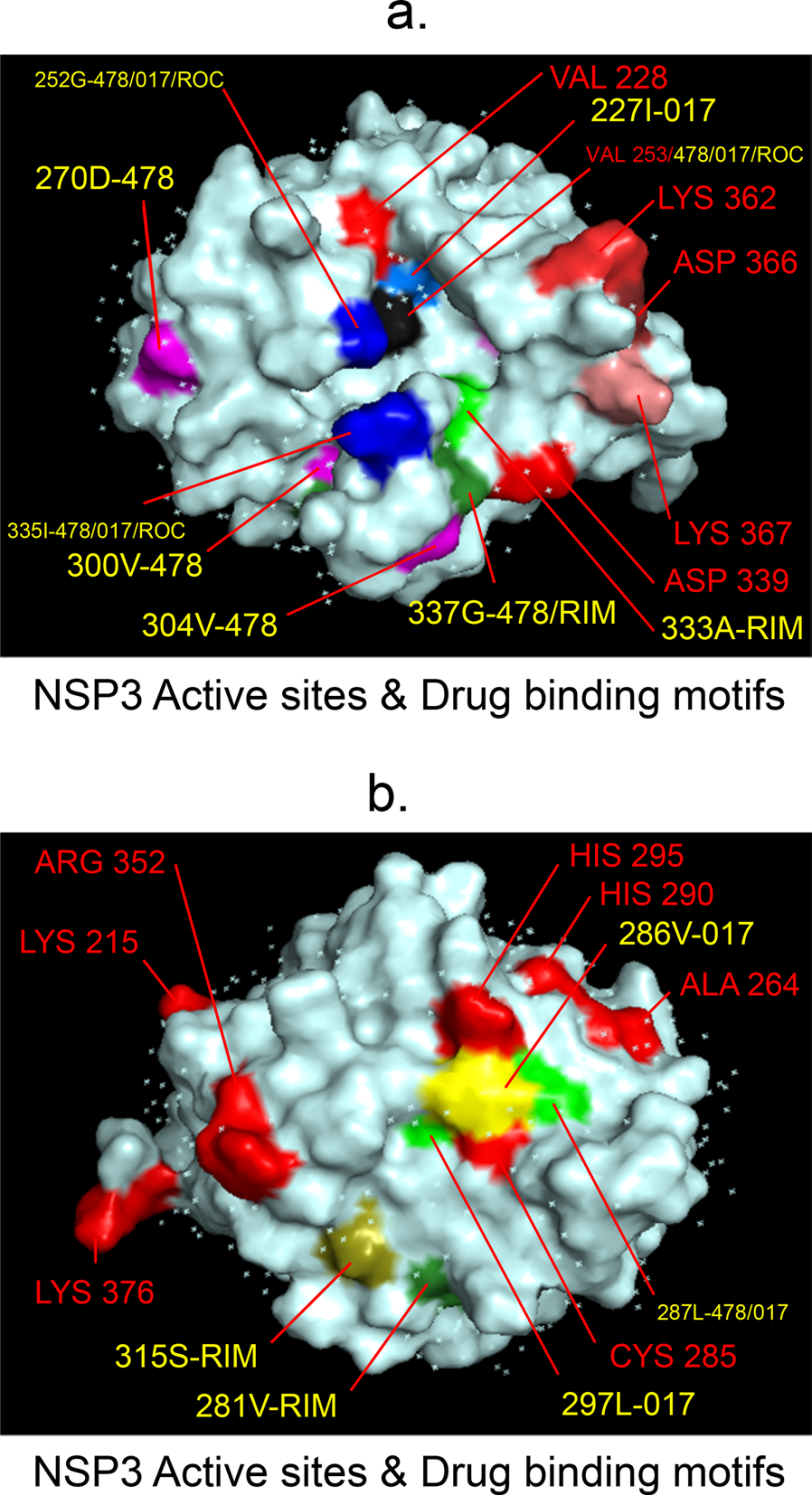
Active site residues & drug binding motifs of NSP3.** a**, **b **Two different surfaces showing drug binding motifs in close association with active site residues of the enzyme. Here Anprenavir, Darunavir and Saquinavir targeted active site residue VAL253 in a pocket. 478-Amprenavir; 017-Darunavir; *RIM* Rimantadine, *ROC* Saquinavir

**Fig. 13:**
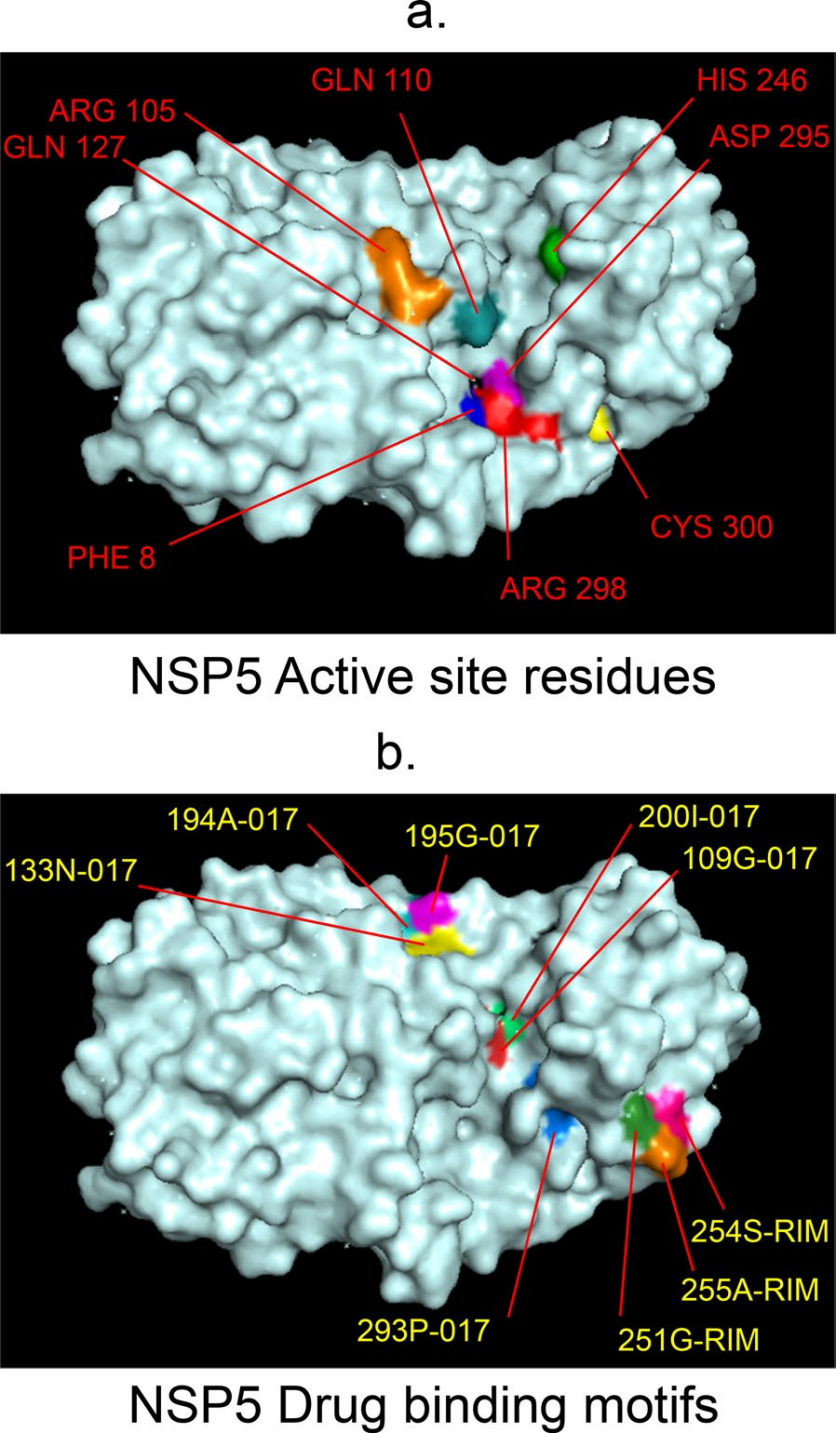
Active site residues & drug binding motifs of NSP5.** a** Position of active site residues within and near a pocket. **b** 017 & RIM targeted residues closely associated with that pocket which accounts for inhibition of active site functioning. 478-Amprenavir; 017-Darunavir; *RIM* Rimantadine, *ROC* Saquinavir

**Fig. 14:**
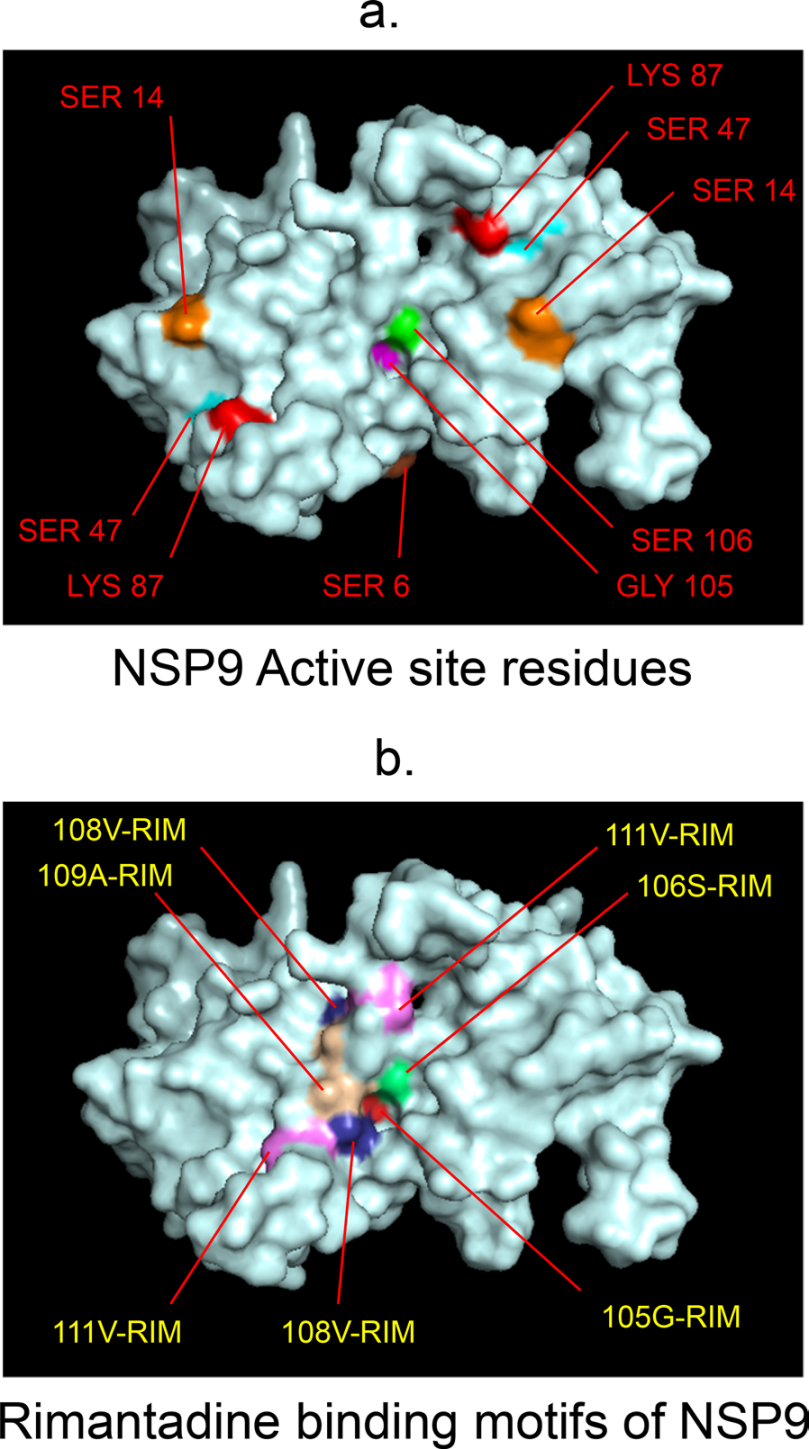
Active site residues & drug binding motifs of NSP9.** a** Surface view showing position of active site residues. **b** Only Rimantadine showed numerous inhibitory binding. 105G and 106S active residues were targeted by RIM. *RIM* Rimantadine

**Fig. 15:**
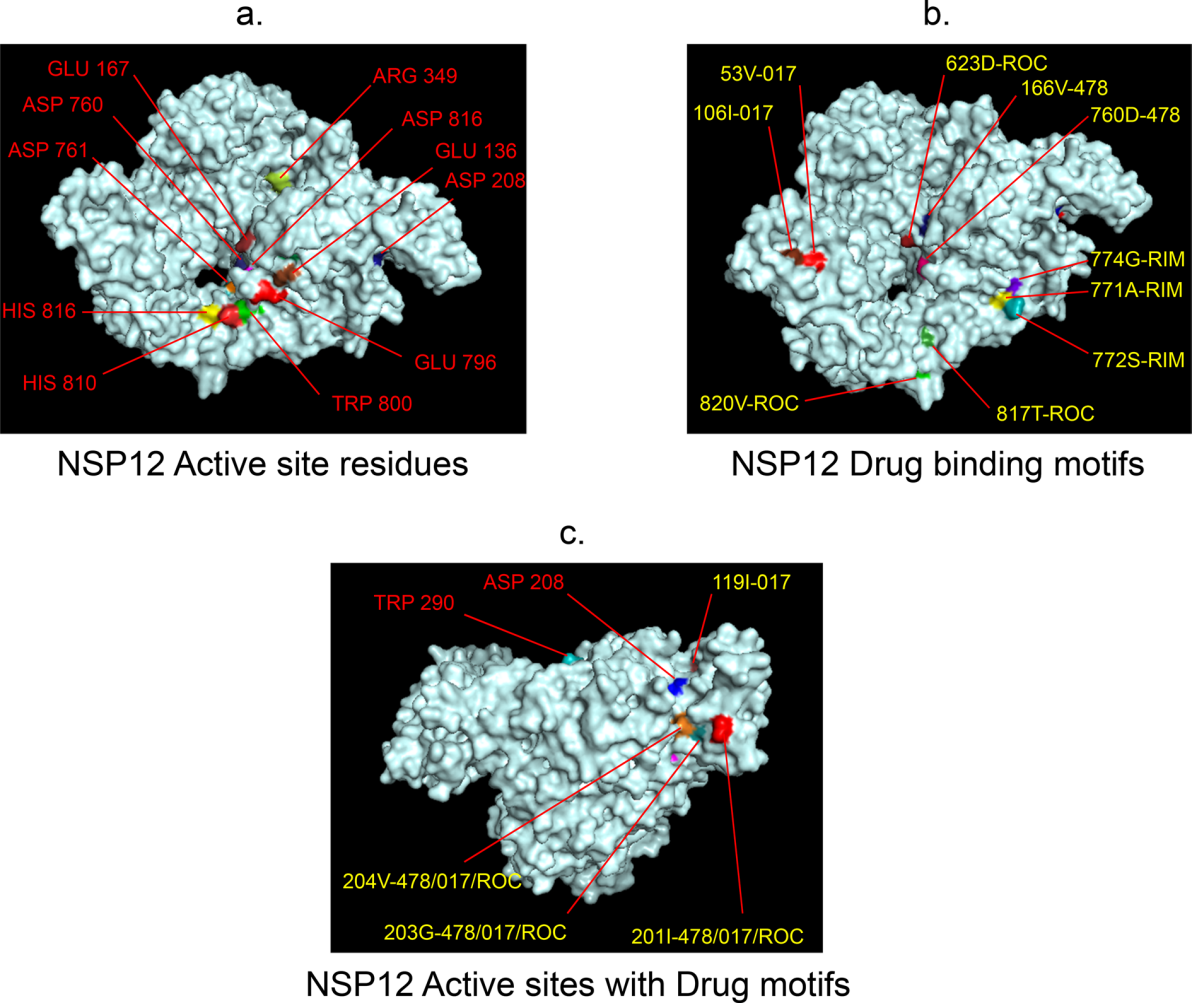
Active site residues & drug binding motifs of NSP12.** a** Position of active site residues. **b**, **c** Different surfaces showing 478, 017, RIM and ROC binding interfaces or residues. 478-Amprenavir; 017-Darunavir; *RIM* Rimantadine, *ROC* Saquinavir

**Fig. 16:**
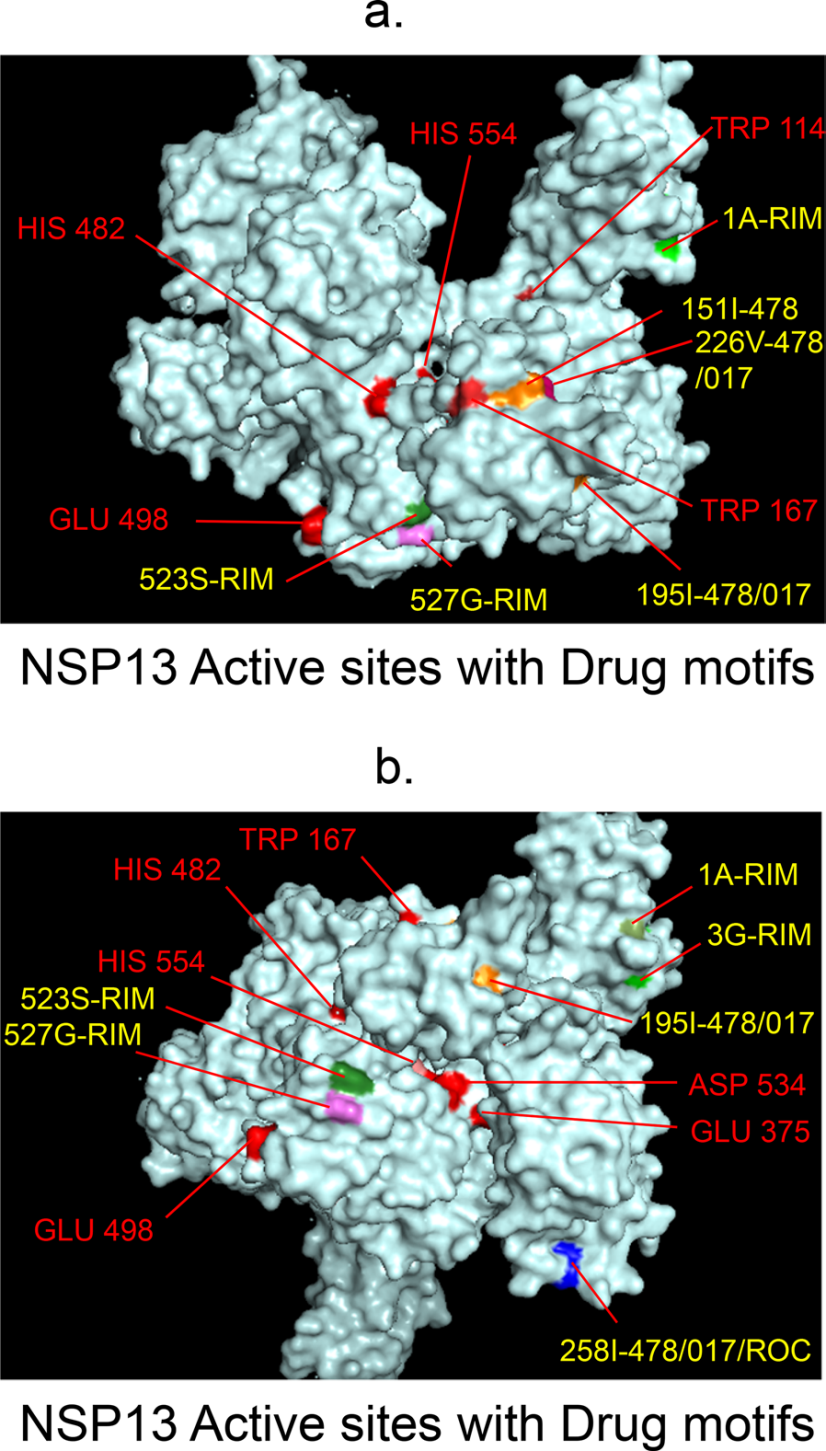
Active site residues & drug binding motifs of NSP13.** a**, **b** Position of active site residues and drug binding motifs in surface presentation. 478-Amprenavir; 017-Darunavir; *RIM* Rimantadine, *ROC* Saquinavir

**Fig. 17:**
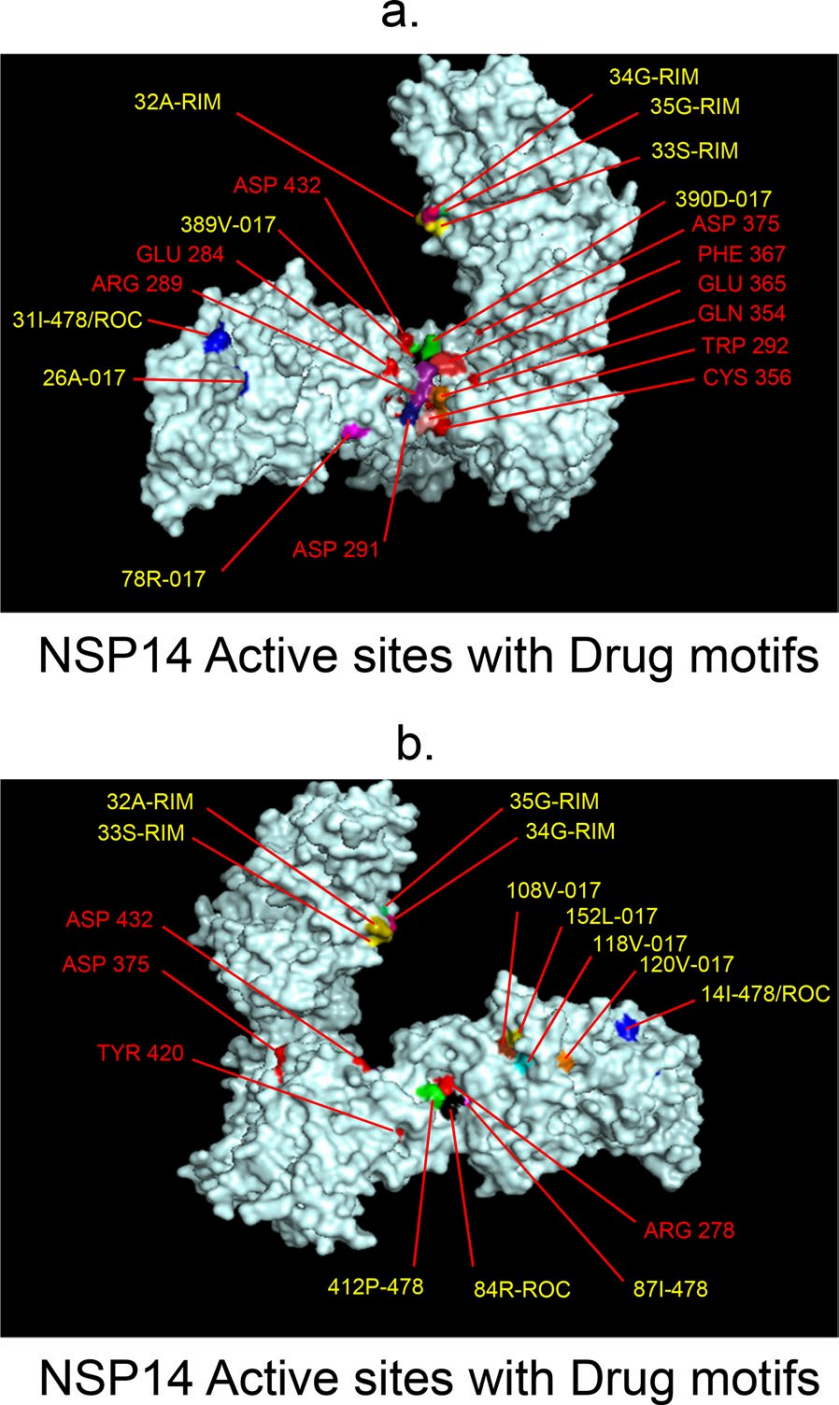
Active site residues & drug binding motifs of NSP14.** a**, **b** Position of active site residues and drug binding interfaces in surface presentation. 478-Amprenavir; 017-Darunavir; *RIM* Rimantadine, *ROC* Saquinavir

**Fig. 18:**
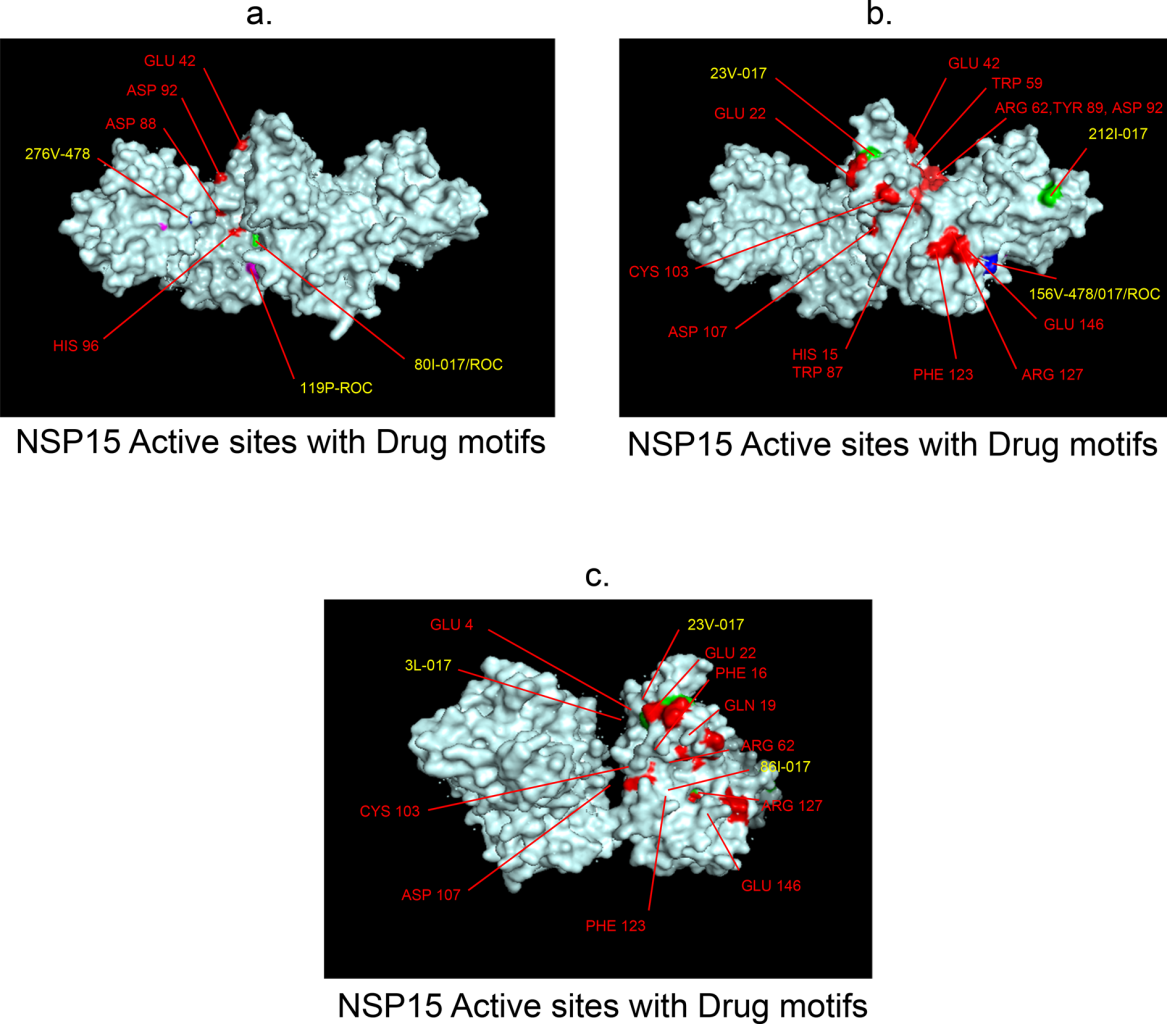
Active site residues & drug binding motifs of NSP15.** a–c** Different surface projections of NSP15 showing positions of active residues and drug binding motifs. 478-Amprenavir; 017-Darunavir; *RIM* Rimantadine, *ROC* Saquinavir

**Fig. 19:**
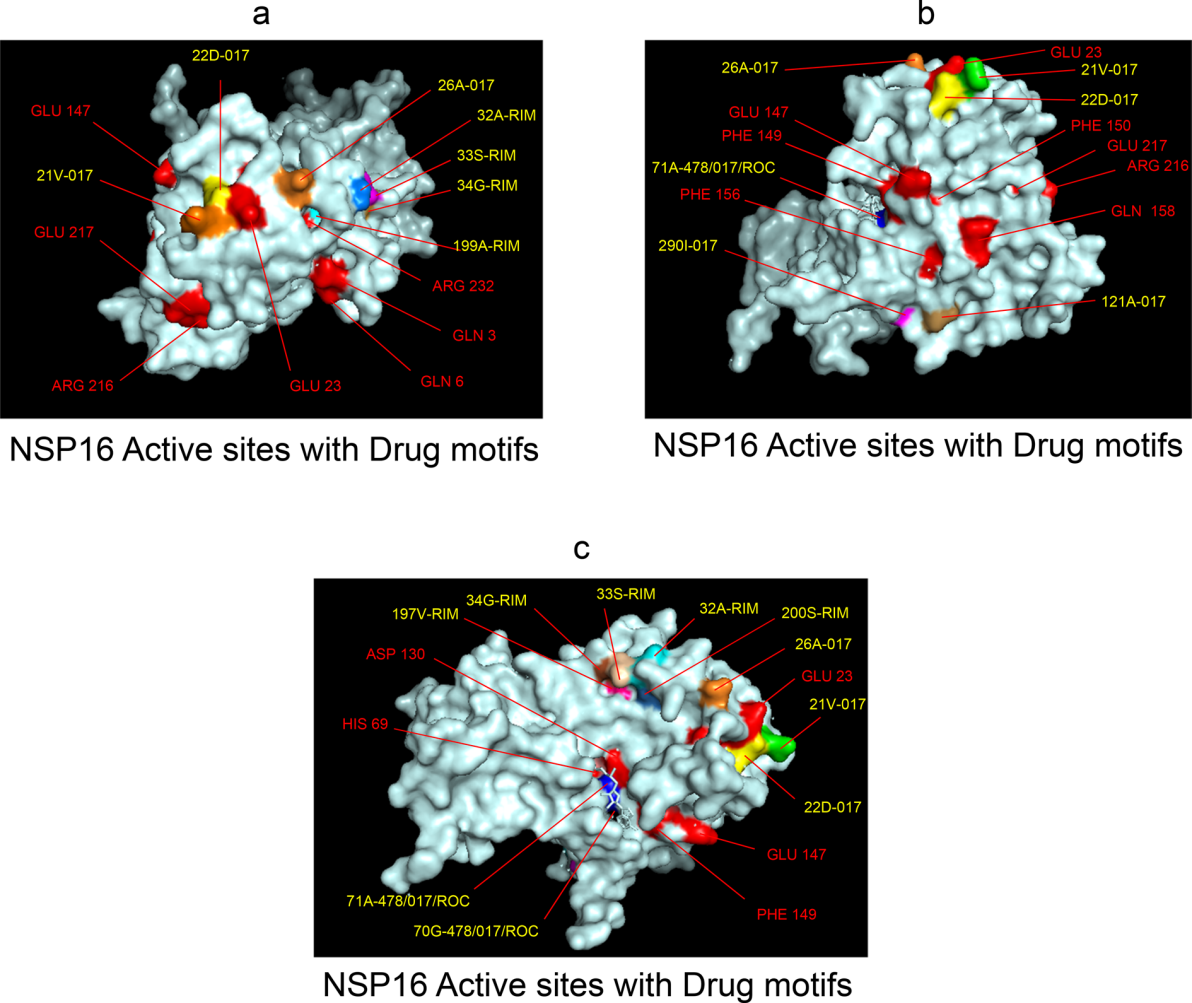
Active site residues & drug binding motifs of NSP16.** a**–**c** Different surface projections showing inhibitory association of drug binding motifs with active site residues of the enzyme. 478-Amprenavir; 017-Darunavir; *RIM* Rimantadine, *ROC* Saquinavir

Among the twelve proteins studied, eight were key enzymes involved in viral replication, transcription and life cycle processes. Hence, the study was further extended to provide insight whether the binding motifs of the selected drugs were significant in inhibiting these enzymes possibly by intercepting active sites of those enzymes. Active sites of enzymes are surface regions that are highly conserved and involved in catalysis or substrate binding. In this study, active sites of SARS-CoV-2 enzymes were predicted by a web server, GASS-WEB (http://gass.unifei.edu.br/) that uses Genetic Active Site Search based on genetic algorithms [51]. Active site residues and the drug binding interfaces of the four drugs viz. Amprenavir (478), Darunavir (017), Rimantadine (RIM) and Saquinavir (ROC) were presented in surface topography presentations of each of the enzymes and were analyzed for their inhibitory association. Results revealed that active site residues of the papain- like protease NSP3 were in close association with drug binding motifs of Amprenavir (270D, 252G, 253 V, 335I, 300 V, 304 V, 287L), Darunavir (252G, 227I, 253 V, 335I, 286 V, 297L, 287L), Rimantadine (337G, 333A, 315S, 281 V) and Saquinavir (252G, 253 V, 335I) (Fig. 12, Table 14). Active sites of protease NSP5 were closely apposed to Darunavir (133 N, 194A, 195G, 200I, 109G, 293P) and Rimantadine (254S, 255A, 251G) binding residues (Fig. 13, Table 14). NSP9 active sites were exclusively targeted by Rimantadine (108 V, 109A, 111 V, 106S, 105G) (Fig. 14, Table 14). RNA polymerase NSP12 active sites were targeted by Amprenavir (166 V, 760D, 203G, 204 V, 201I), Darunavir (53 V, 106I, 119I, 203G, 204 V, 201I), Rimantadine (774G, 771A, 772S) and Saquinavir (623D, 817 T, 820 V, 203G, 204 V, 201I) (Fig. 15, Table 14). The helicase NSP13 active residues were targeted by Amprenavir (195I, 151I, 226 V, 258I), Darunavir (195I, 226 V, 258I), Rimantadine (1A, 3G, 523S, 527G) and Saquinavir (258I) (Fig. 16, Table 14). Exoribonuclease NSP14 active sites were closely apposed to Amprenavir (31I, 14I, 87I, 412P), Darunavir (389 V, 26A, 78R, 390D, 108 V, 152L, 118 V, 120 V), Rimantadine (32A, 34G, 35G, 33S) and Saquinavir (31I, 14I, 84R) binding residues (Fig. 17, Table 14). On the other hand, endonuclease NSP15 active sites were targeted by Amprenavir (276 V, 156 V), Darunavir (80I, 23 V, 212I, 156 V, 3L, 86I), and Saquinavir (119P, 80I, 156 V) (Fig. 18, Table 14). Finally, methyltransferase NSP16 active site residues were targeted by Amprenavir (71A, 70G), Darunavir (21 V, 22D, 26A, 71A, 290I, 121A, 200S), Rimantadine (32A, 33S, 34G, 199A, 197 V, 200S) and Saquinavir (71A, 70G) (Fig. 19, Table 14). Close association of drug binding motifs with the active sites indicated that these would interfere with catalytic activity and substrate binding of the enzymes.

Previously, several drug repurposing analysis were performed by several groups to find potential drug inhibitors like sirolimus, dactinomycin, mercaptopurine, melatonin, toremifene, emodin, zotatifin, ternatin-4, hydroxychloroquine, clemastine, Atazanavir, remdesivir, efavirenz, Ritonavir, dolutegravir, carfilzomib, cyclosporine A, azithromycin, favipiravir, Ribavirin, galidesivir and many others against SARS-CoV-2 proteins but their efficacy is questionable in treating and curing COVID-19 patients [52–57].

## Conclusion

The findings strongly suggested that among the fourteen anti-viral drugs predicted and analyzed, six drugs significantly targeted twelve SARS-Cov2 non structural proteins and specifically the key enzymes. Considering the binding parameters it can be concluded that combination of Darunavir (DB01264), Amprenavir(DB00701) and Rimantadine(DB00478) or Saquinavir (DB01232), Amprenavir (DB00701) and Rimantadine (DB00478) or all the four drugs together can potentially bind and inhibit the cellular activities of these proteins that are essential for viral replication and life cycle. Using anti-viral drug has great advantage in that these have specific target and less or no similar binding partners like Rimantadine had no other binding partners other than SARS-Cov-2 NSPs (Tables 1, 2, 3, 4, 5, 6, 7, 8, 9, 10 and 11). Finally, these predicted drug combinations must be clinically tested to save thousands of lives in the vicinity of limited effectiveness of developed vaccines [58, 59].

## Methods

### Key resources table

**Table:** 

Resource	Source	Identifier
Analyzed data		
SARS-CoV-2 NSP1 3D-structure	[25]	PDB ID: 7K3N
SARS-CoV-2 NSP3 3D-structure	[26]	PDB ID: 6WEY
SARS-CoV-2 NSP5 3D-structure	[27]	PDB ID: 6M03
SARS-CoV-2 NSP7-8 complex 3D-structure	[28]	PDB ID: 7JLT
SARS-CoV-2 NSP9 3D-structure	[29]	PDB ID: 6W4B
SARS-CoV-2 NSP10 3D-structure	[30]	PDB ID: 6ZCT
SARS-CoV-2 NSP7-8-12 complex 3D-structure	[31]	PDB ID: 6M71
SARS-CoV-2 NSP13 3D-structure	[32]	PDB ID: 7NIO
SARS-CoV-2 NSP14 3D-structure	[33]	PDB ID: 5C8S
SARS-CoV-2 NSP15 3D-structure	[34]	PDB ID: 6VWW
SARS-CoV-2 NSP16-10 complex 3D-structure	[35]	PDB ID: 7BQ7
Web server		
DrReposER	[37]	http://27.126.156.175/drreposed/
GASS-WEB	[51]	http://gass.unifei.edu.br/

DrReposERhas been used to find binding interfaces or 3D-motifs of target proteins (PDB ID: 7K3N, 6WEY, 6M03, 7JLT, 6W4B, 6ZCT, 6M71, 7NIO, 5C8S, 6VWW and 7BQ7) for all possible drugs. The program uses SPRITE and ASSAM web servers to find amino acid side chains. Drug ReposER compares structurally similar side chain arrangements from PDB repository and assign hit results for different drug targets in the query PDB ID [37].

GASS-WEB has been used to predict active sites of SARS-CoV-2 enzymes (NSP3, NSP5, NSP9, NSP12, NSP13, NSP14, NSP15 and NSP16) considered in this study. It uses genetic algorithms to find active sites of enzymes that are meant for catalytic activity or substrate binding [51].

## Supplementary Information

**Additional file 1: S1.** List of drug binding hits for 7K3N –NSP1.

**Additional file 1: S2.** List of drug binding hits for 6WEY-NSP3.

**Additional file 1: S3.** List of drug binding hits for 6M03 –NSP5.

**Additional file 1: S4.** List of drug binding hits for 7JLT-NSP7-8.

**Additional file 1: S5.** List of drug binding hits for 6W4B-NSP9.

**Additional file 1: S6.** List of drug binding hits for 6ZCT-NSP10.

**Additional file 1: S7.** List of drug binding hits for 6M71-NSP7-8-12.

**Additional file 1: S8.** List of drug binding hits for 7NIO-NSP13.

**Additional file 1: S9.** List of drug binding hits for 5C8S-NSP14.

**Additional file 1: S10.** List of drug binding hits for 6VWW-NSP15.

**Additional file 1: S11.** List of drug binding hits for 7BQ7-NSP16-10.

## Data Availability

Not applicable.
